# Supplementary material for: Mitochondrial pyruvate metabolism in club cells drives airway inflammation
Source: Stem Cell Reports. 2025 Dec 18;21(1):102742. doi: 10.1016/j.stemcr.2025.102742 (PMC12925956; doi:10.1016/j.stemcr.2025.102742)
Supplement: Document S1. Figures S1–S4 and supplemental methods [file mmc1.pdf]

Stem Cell Reports, Volume *21*

Supplemental Information

Mitochondrial pyruvate metabolism in club cells drives airway inflammation

Jianhai Wang, Chunnan Du, De Hao, Qian Wu, Biyu Gui, Yu Li, Kuan Li, Xue Li, Qiuyang Zhang, Li Li, and Huaiyong Chen

Supplemental Information

Mitochondrial pyruvate metabolism in club cells drives airway inflammation

Jianhai Wang, Chunnan Du, De Hao, Qian Wu, Biyu Gui, Yu Li, Kuan Li, Xue Li, Qiuyang Zhang, Li Li, Huaiyong Chen

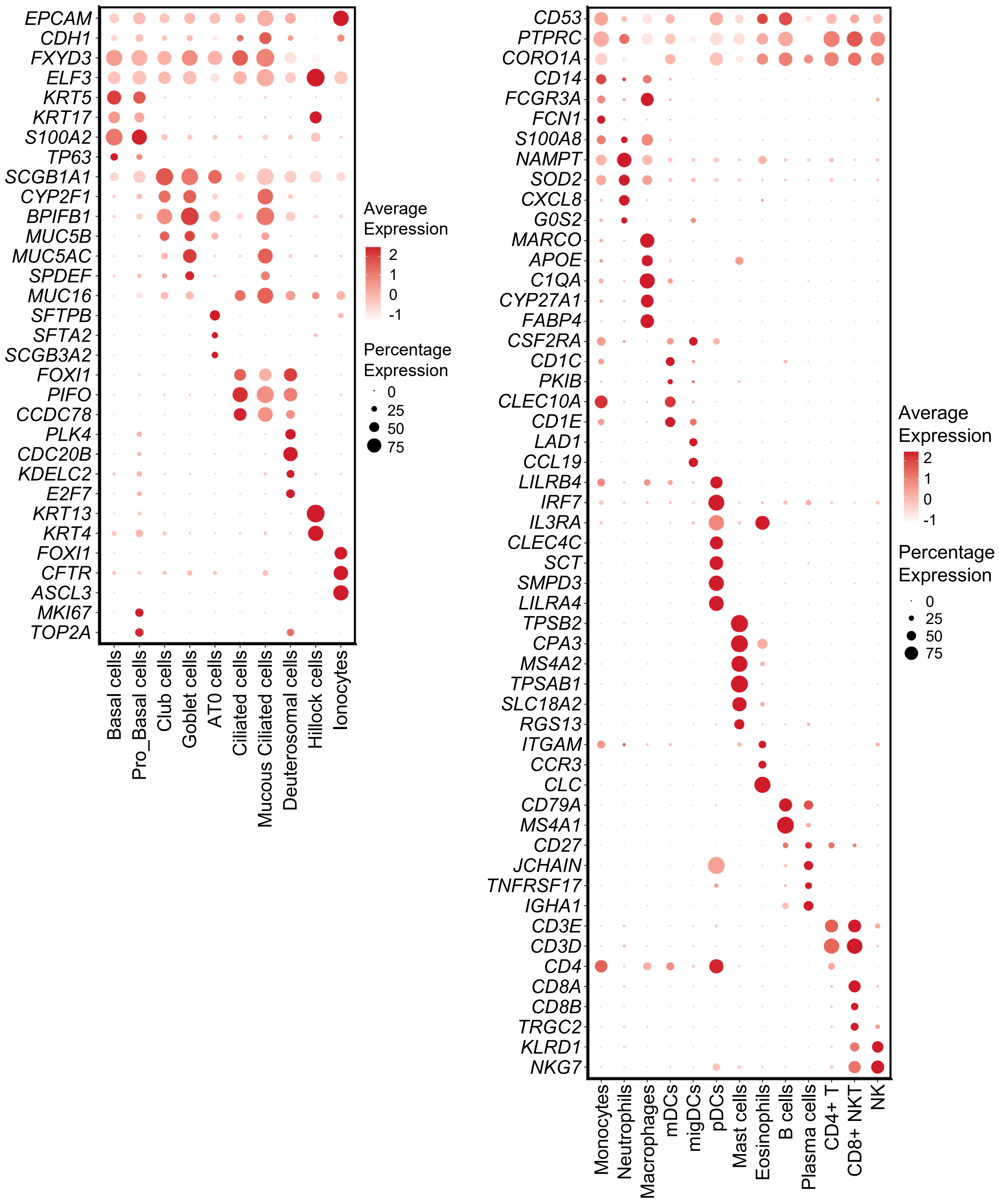

Figure S1. Marker gene expression patterns across distinct cell types

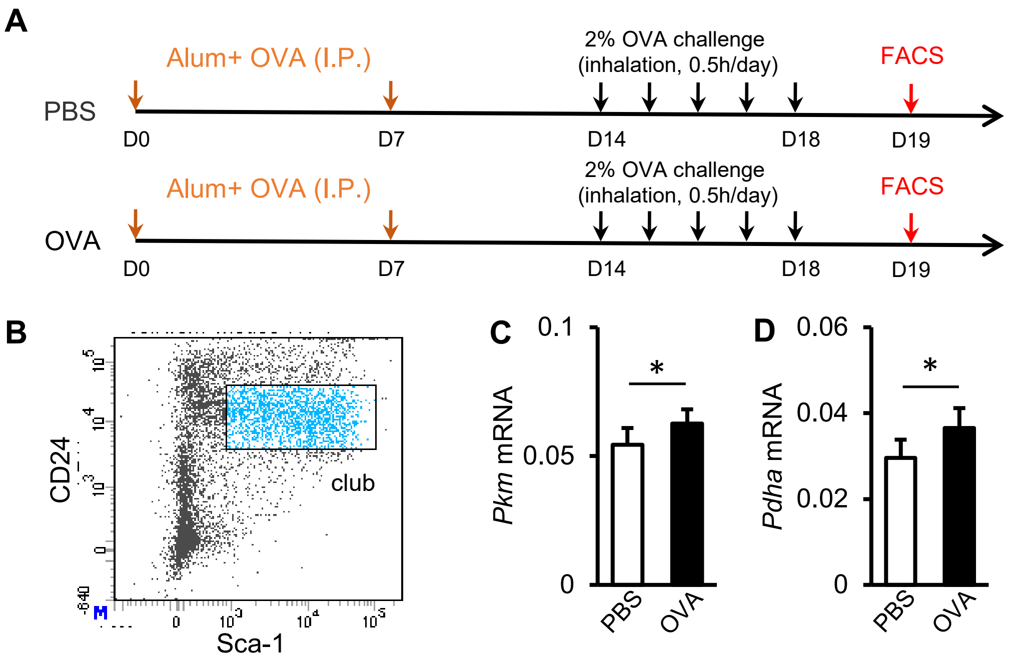

Figure S2. OVA-induced inflammation activates glycolysis and OXPHOS in club cells.

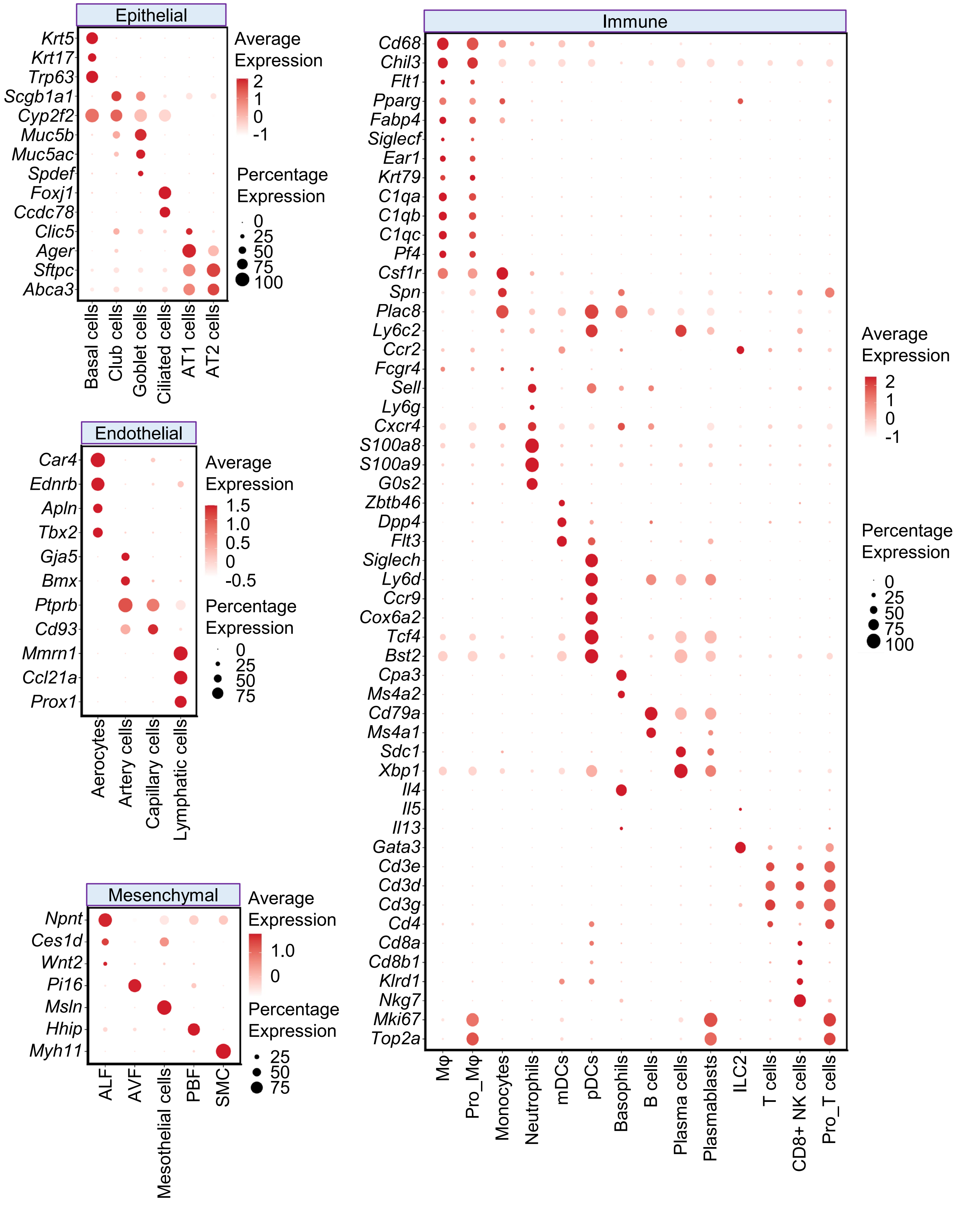

Figure S3. Marker gene expression patterns across distinct cell types.

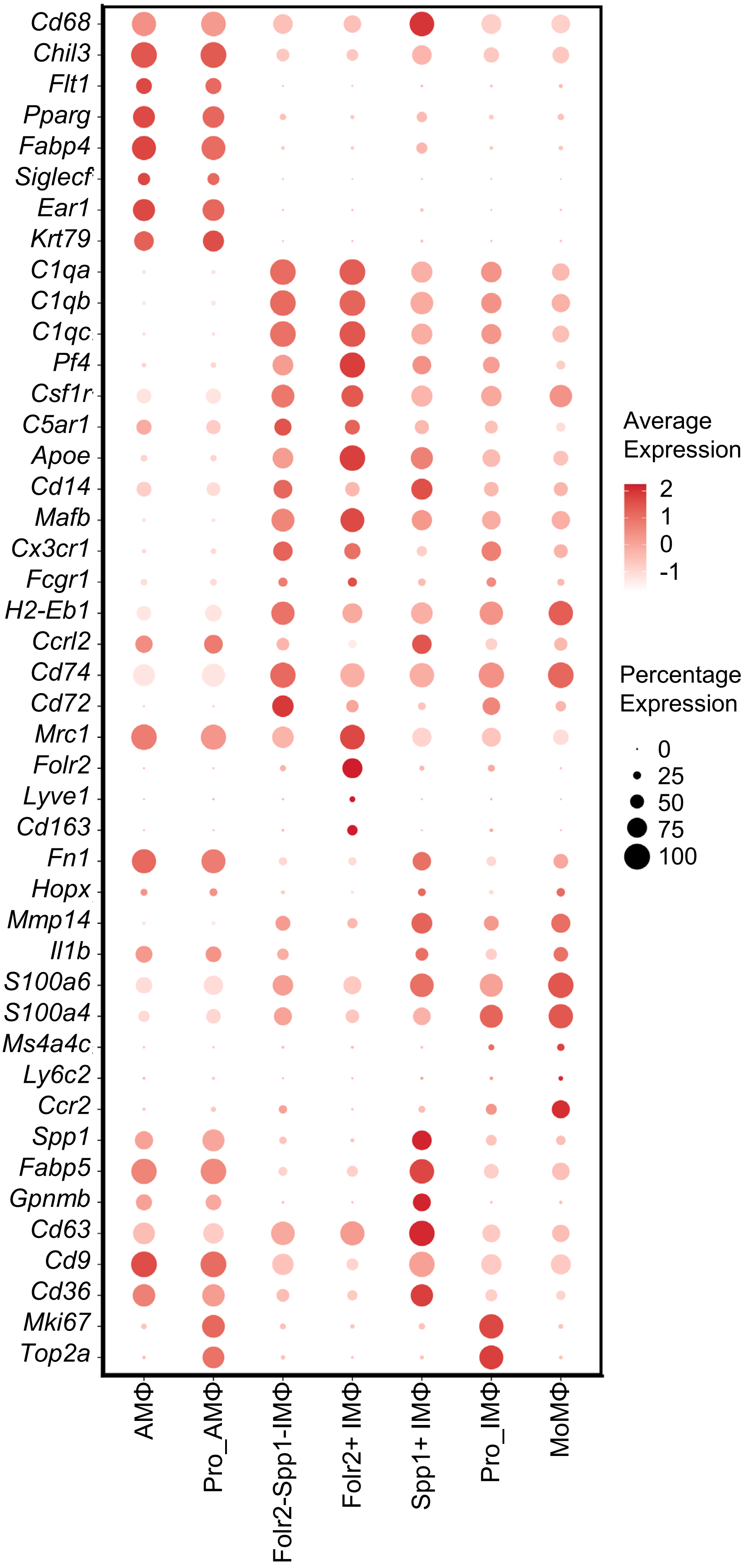

Figure S4. Marker gene expression patterns of macrophages.

Figure S1 Marker gene expression patterns across distinct cell types

The dot plot illustrates the average expression levels (color intensity) and the proportion of positively expressing cells (dot size) for representative marker genes across 23 cell types. The left panel displays marker expression in epithelial-derived cells (e.g., EPCAM, KRT5, SCGB1A1, FOXJ1, PIFO), while the right panel shows marker expression in immune cell populations (e.g., CD14, FCGR3A, MARCO, IL3RA, CD3D, CD79A, NKG7). Color indicates the average expression value.

Figure S2 OVA-induced inflammation activates glycolysis and OXPHOS in club cells

(A) Schematic illustration of the OVA-induced asthma model. Mice were sensitized by intraperitoneal injection of alum/OVA on days 0 and 7, followed by exposure to 2% OVA aerosol for 30 minutes per day from days 14 to 18. Lung tissues were harvested 24 hours after the final challenge (day 19).

(B) Representative flow cytometry plot showing the gating strategy for isolating $CD24^{+}Sca\text{-}1^{+}$ club cells from lung tissue.

(C - D) qPCR was performed on sorted club cells from OVA-treated mice compared with PBS controls ($P < 0.05$, n = 5 per group) to assess *Pkm* mRNA (C) and *Pdha* mRNA expression (D) levels. Data are presented as mean ± SD; statistical significance was determined using an unpaired two-tailed Student's t-test.

Figure S3 Marker gene expression patterns across distinct cell types

Dot plot showing expression of canonical marker genes in epithelial, endothelial, mesenchymal, and immune cell types. Dot color reflects average expression level, while size indicates the percentage of cells expressing each gene. Marker genes distinguish key subtypes such as club, goblet, and AT2 cells; endothelial and fibroblast subpopulations; and diverse immune cell types including macrophages, neutrophils, dendritic cells, and T/B cells.

Figure S4 Marker gene expression patterns of macrophages

A dot plot of marker genes across macrophage subpopulations reveals distinct transcriptional signatures. The color gradient represents the average expression level (Average Expression), with lighter to darker shades indicating increasing expression.

The size of each dot indicates the percentage of cells within the cluster that positively express the corresponding gene.

Supplemental experimental methods

The OVA-induced model was conducted according to the protocol described in the main text, with identical dose, timeline, and treatments. Cell sorting and molecular assays were performed as described in the “Flow cytometry” and “qPCR” sections of the main text. The qPCR target genes were *Pkm* and *Pdha*. Relative expression was calculated using the $2^{-\Delta\Delta Ct}$ method with Gapdh as the reference gene. Data are presented as mean ± SD. Group comparisons were performed using unpaired two-tailed Student’s t-tests with a significance threshold of $P < 0.05$. Biological replicates were n = 5 per group.
