# Supplementary material for: Mitochondrial pyruvate metabolism in club cells drives airway inflammation
Source: Stem Cell Reports. 2025 Dec 18;21(1):102742. doi: 10.1016/j.stemcr.2025.102742 (PMC12925956; doi:10.1016/j.stemcr.2025.102742)
Supplement: Document S2. Article plus supplemental information [file mmc2.pdf]

STEM CELL REPORTS
Article

OPEN ACCESS

Mitochondrial pyruvate metabolism in club cells drives airway inflammation

Jianhai Wang,[1,3,6] Chunnan Du,[1,6] De Hao,[1,6] Qian Wu,[1,6] Biyu Gui,[1] Yu Li,[2] Kuan Li,[2] Xue Li,[2] Qiuyang Zhang,[2] Li Li,[1,4,*] and Huaiyong Chen[1,2,3,4,5,7,*]
[1]Department of Respiratory Medicine, Haihe Clinical School, Tianjin Medical University, Tianjin 300350, China
[2]Department of Basic Medicine, Haihe Hospital, Tianjin University, Tianjin 300350, China
[3]Tianjin Key Laboratory of Lung Regenerative Medicine, Tianjin 300350, China
[4]Key Research Laboratory for Infectious Disease Prevention for State Administration of Traditional Chinese Medicine, Tianjin Institute of Respiratory Diseases, Tianjin 300350, China
[5]Key Laboratory of Medical Rescue Key Technology and Equipment, Ministry of Emergency Management, Beijing 100028, China
[6]These authors contributed equally
[7]Lead contact
*Correspondence: lily20060718@163.com (L.L.), huaiyong.chen@foxmail.com (H.C.)
https://doi.org/10.1016/j.stemcr.2025.102742

SUMMARY

Asthma is a chronic inflammatory airway disease characterized by defective epithelial repair, resulting from metabolic dysregulation in facultative progenitor cells. Here, we investigate how pyruvate metabolism in airway club cells controls epithelial differentiation and allergic airway inflammation. Single-cell transcriptomics revealed elevated glycolytic activity in club and goblet cells from patients with asthma. In an ovalbumin (OVA)-induced asthma model, conditional deletion of *Mpc2*—but not *Ldha*—in club cells impaired club-to-goblet cell differentiation, reduced CLCA3 and *Foxa3* expression, and attenuated eosinophilic inflammation and *Il-13* expression. *Mpc2* loss increased *Cxcl17* expression in club cells, promoting *Cxcl17-Cxcr4* signaling with alveolar macrophages that suppressed CCL17-mediated type 2 inflammation. Neutralizing CCL17 phenocopied the *Mpc2* knockout by reducing airway inflammation and goblet cell differentiation. These findings reveal a metabolic-immune crosstalk underlying asthma pathogenesis and identify mitochondrial pyruvate metabolism as a therapeutic target to limit epithelial remodeling and type 2 inflammation.

INTRODUCTION

Asthma is a common chronic airway disease that affects over 300 million people worldwide, with projections estimating that it will reach nearly 400 million by 2025 (Barcik et al., 2020). The characteristic pathological features of asthma include chronic airway inflammation, smooth muscle hypertrophy, goblet cell hyperplasia, and airway mucosal fibrosis, all of which contribute to airway remodeling (Varricchi et al., 2024). Current clinical management relies primarily on anti-inflammatory drugs to control inflammation, and curative treatments remain unavailable, especially for moderate-to-severe refractory asthma. Recent studies suggest that impaired repair of the airway epithelium following injury is a key pathophysiological mechanism driving persistent and exacerbated inflammation in asthma (Bradding et al., 2024; Zhao et al., 2025). However, the molecular mechanisms underlying defective epithelial repair remain largely unclear, particularly regarding the metabolic regulation of airway epithelial progenitor cell function.

Airway epithelial repair mainly depends on endogenous pulmonary stem/progenitor cells (Heijink et al., 2020; Shao et al., 2024). Among them, club cells are widely distributed throughout both large and small airways, possess both secretory and regenerative functions, and have been identified as facultative progenitor cells in the airway epithelium (Bondeelle et al., 2025; Wang et al., 2024). Club cells can differentiate into ciliated and goblet cells, thus playing a crucial role in maintaining epithelial integrity (Wang et al., 2020a). In recent years, metabolic reprogramming has emerged as a key area of research in the regulation of stem cell function (Ly et al., 2020; Wang et al., 2023). Glucose, a major energy source, is metabolized through two distinct pyruvate pathways—lactate production and oxidative phosphorylation—to regulate stem cell proliferation and differentiation (Li et al., 2019, 2025). It has been reported that knockout of mitochondrial pyruvate carrier (MPC) in airway basal cells impedes pyruvate entry into the tricarboxylic acid cycle, reduces oxidative phosphorylation activity, and forces cells to rely on glycolysis for energy production (Li et al., 2025). This metabolic shift leads to the accumulation of lactate and cytosolic pyruvate, affecting acetyl-CoA production via ATP-citrate lyase, reducing histone acetylation, altering gene transcription, and ultimately enhancing proliferation while impairing differentiation of basal cells (Li et al., 2025). Other studies have shown that enhanced lactate metabolism promotes intestinal stem cell proliferation, whereas oxidative phosphorylation favors hair follicle stem cell differentiation (Flores et al., 2017; Hamanaka et al., 2013). Our previous findings also indicated significant alterations in glucose metabolism within the airways during asthma pathogenesis, suggesting a potential role for metabolic pathways in airway repair and inflammatory processes, although the precise mechanisms remain to be elucidated (Li et al., 2019).

© 2025 The Author(s).
Published by Elsevier Inc. on behalf of International Society for Stem Cell Research.
This is an open access article under the CC BY license (http://creativecommons.org/licenses/by/4.0/).

Table 1. Search strategies and results of GEO data

	Search expression	Results
1	((((scRNA) OR single cell)) AND (lung)) AND asthma	116 (GSE164015, GSE193816)
2	(((((((healthy) OR control)) AND human) AND ((lung) OR pulmonary)) AND (((single cell) OR scRNA) OR single-cell))) AND ((brushes) OR brush)	5 (GSE193816, GSE143868)

This table outlines the search expressions used to identify relevant single-cell RNA sequencing (scRNA-seq) datasets. The first query targeted asthma-related lung single-cell datasets and returned 116 results, including GSE164015 and GSE193816. The second query focused on healthy human lung or pulmonary samples collected via airway brushing, yielding 5 results, including GSE193816 and GSE143868.

Despite previous evidence linking club cell dysfunction to asthma progression and initial insights into the role of glucose metabolism in club cell function, the specific impact of distinct pyruvate metabolic pathways—namely lactate production versus oxidative phosphorylation—on club cell proliferation and differentiation has not been systematically studied (Li et al., 2022; Zhu et al., 2019). Furthermore, whether pyruvate metabolic reprogramming contributes to persistent airway inflammation and remodeling in asthma remains unclear. This gap in knowledge has hindered the development of novel therapeutic strategies targeting airway regeneration in asthma.

To address these critical questions, we employed an ovalbumin (OVA)-induced allergic airway inflammation model combined with conditional gene knockout technology and single-cell RNA sequencing (scRNA-seq) to systematically investigate the role of pyruvate metabolic pathways in club cells during asthma pathogenesis. Specifically, we selectively deleted *Ldha*, a key gene in the lactate pathway, and *Mpc2*, a key gene in the mitochondrial oxidative phosphorylation pathway, in club cells. We examined how these distinct metabolic routes influence club cell proliferation, differentiation, and epithelial repair capacity and further analyzed their effects on airway inflammation and remodeling. Our findings aim to elucidate the mechanistic link between metabolic dysregulation in airway progenitor cells and asthma pathology and may provide a theoretical foundation for developing metabolism-targeted therapies for asthma.

RESULTS

Glycolysis is enhanced in club cells of asthma patients

To investigate changes in glucose metabolism in airway epithelial cells from asthma patients, we retrieved public human scRNA-seq datasets using two query strategies (Table 1). Basic dataset information is shown in Figure 1A. After data import, quality control, and dimensionality reduction using the Seurat package, 55,724 cells from airway brushings (15,617 from healthy controls and 40,107 from asthma patients) were annotated into 23 cell types based on canonical marker gene expression (Figure 1B). The expression profiles of marker genes for each cell type are presented in Figure S1. To assess cellular functional differences, we conducted gene set variation analysis (GSVA) on epithelial cells using all 50 hallmark gene sets from the Mouse Molecular Signatures Database. Statistical analysis of GSVA scores revealed significantly enhanced glycolytic activity in both club cells and goblet cells from asthma patients compared to healthy controls (Figure 1C). To confirm glycolytic activation *in vivo*, we isolated club cells from OVA-induced asthmatic and control mice. Quantitative polymerase chain reaction (qPCR) analysis demonstrated significant upregulation of *Pkm* (pyruvate kinase M) and *Pdha* (pyruvate dehydrogenase E1 α-subunit) mRNA expression in OVA-treated mice (Figure S2), indicating that both glycolytic and pyruvate oxidative pathways are enhanced in club cells during allergic airway inflammation.

The oxidative phosphorylation pathway of pyruvate promotes club-to-goblet cell differentiation in mice

After entering cells, glucose is converted to pyruvate via glycolysis. Pyruvate can be further metabolized through two distinct routes: (1) conversion to lactate by lactate dehydrogenase (LDH) or (2) mitochondrial import via the MPC 1/2 (MPC1/2) for oxidative phosphorylation. To examine the fate of glycolytic pyruvate, we generated *Scgb1a1-CreER; Mpc2*$^{f/f}$ conditional knockout mice (*Mpc2CKO*) to block mitochondrial entry of pyruvate (Figure 2A). qPCR confirmed a marked reduction in *Mpc2* mRNA in fluorescence-activated cell sorting (FACS)-sorted club cells from *Mpc2CKO* (Figures 2B–2D), and immunofluorescence staining further demonstrated efficient depletion of MPC2 protein in club cells in lung tissue (Figure 2E). White dashed circles delineate club-cell regions, and weak MPC2 signals outside these areas likely originate from adjacent myofibroblasts (Figure 2E).

Consistent with impaired mitochondrial pyruvate utilization in *Mpc2CKO* club cells, *Pkm* and *Pdha* mRNA levels were also decreased relative to control (*Mpc2*$^{f/f}$) (Figures 2F and 2G). We next challenged mice in an OVA-induced allergic airway inflammation model. Tamoxifen (Tam) was administered to activate Cre recombinase prior to OVA aerosol challenge (Figure 2H), and bronchoalveolar lavage fluid (BALF) and lung tissues were collected for analysis. Compared to control mice, the *Mpc2CKO* group showed a significant reduction in the proportion of $CLCA3^{+}$ goblet cells in the lung (Figure 2I). qPCR analysis revealed significantly lower

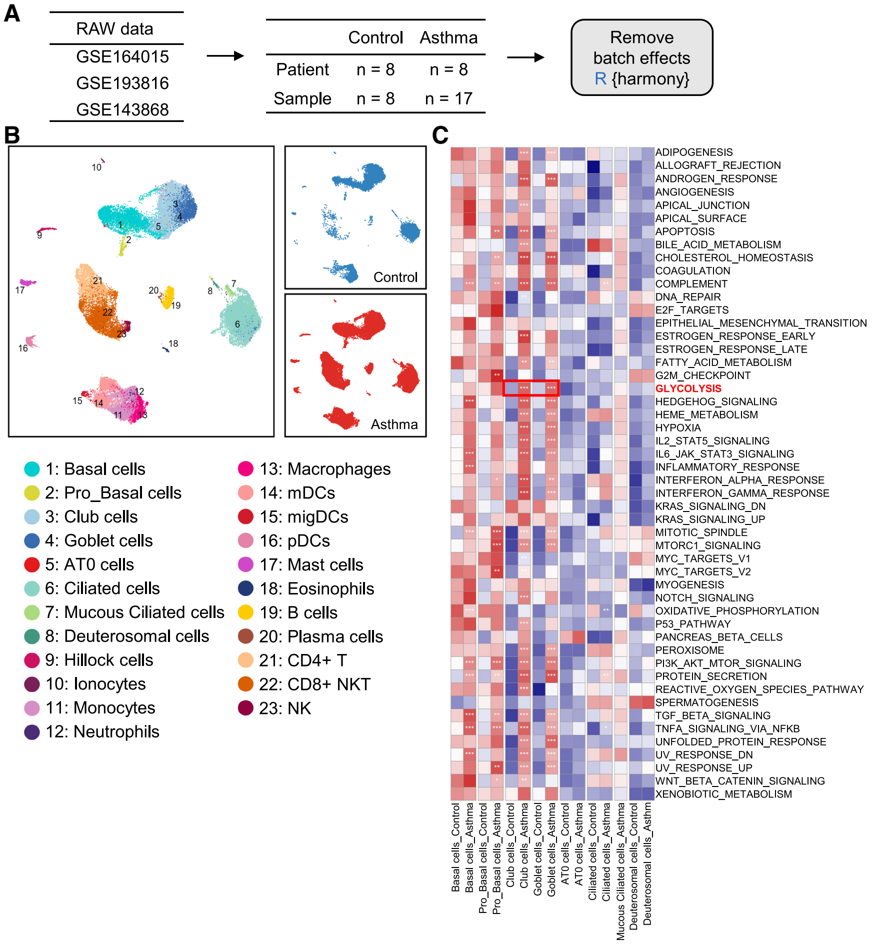

Figure 1. The glycolysis is significantly enhanced in club cells from asthma patients

(A) Overview of three publicly available scRNA-seq datasets (GSE164015, GSE193816, and GSE143868) including samples from asthma patients ($n = 8$) and healthy controls ($n = 8$), totaling 17 samples. Batch effects were corrected using the Harmony algorithm in R.

(B) UMAP visualization of 55,724 airway cells. A total of 23 distinct cell types were annotated, including basal cells, goblet cells, monocytes, dendritic cells (DCs), and neutrophils. Cells from healthy controls and asthma patients are shown in different colors.

(C) GSVA using hallmark gene sets reveals functional differences between asthma and control groups, highlighting elevated glycolysis in club and goblet cells in asthma.

mRNA levels of *Clca3* (Figure 2J) and the goblet cell transcription factor *Foxa3* (Figure 2K). These results indicate that disruption of pyruvate oxidative phosphorylation inhibits club-to-goblet cell differentiation. Analysis of BALF revealed reduced total inflammatory cell counts in the *Mpc2CKO* group, particularly eosinophils and macrophages (Figure 2M). Lung tissue expression of *Il-13* was also significantly decreased (Figure 2L). Together, these findings suggest that blocking oxidative phosphorylation in club cells not only impairs their differentiation but also mitigates airway inflammation.

Loss of LDHA-dependent lactate production does not significantly alter club-to-goblet cell differentiation

As noted previously, pyruvate can also be converted to lactate (Figure 3A). To explore the role of this pathway, we generated *Scgb1a1-CreER; Ldha$^{f/f}$* (*LdhaCKO*) conditional knockout mice to inhibit lactate production (Figures 3A–3D). These mice were subjected to an OVA-induced airway inflammation model, and BALF and lung tissues were analyzed (Figure 3E). Immunofluorescence staining showed no significant changes in club cell proliferation or goblet cell differentiation (CLCA3$^+$ cells) in the *LdhaCKO* group compared to controls (Figure 3F). Inflammatory cell counts in BALF, including eosinophils, neutrophils, macrophages, and lymphocytes, were not significantly different between groups (Figure 3G). Enzyme-linked immunosorbent assay (ELISA) analysis showed no significant differences in interleukin (IL)-4 or IL-13 levels in BALF between groups (Figures 3H and 3I). These findings indicate that while *Ldha* deletion effectively blocks lactate production in club cells, it does not significantly impact their proliferation, differentiation, or inflammatory responses, suggesting that LDHA-dependent lactate metabolism plays a limited role in club-to-goblet cell differentiation.

Mpc2 deletion remodels the transcriptome of club cells and upregulates *Cxcl17* expression

To explore the possible molecular mechanisms by which pyruvate oxidative phosphorylation affects club cell differentiation and airway inflammation, we performed both scRNA-seq and bulk RNA sequencing (RNA-seq) on lung tissues from control-OVA and *Mpc2CKO-OVA* mice following OVA-induced airway inflammation (Figure 4A). After quality

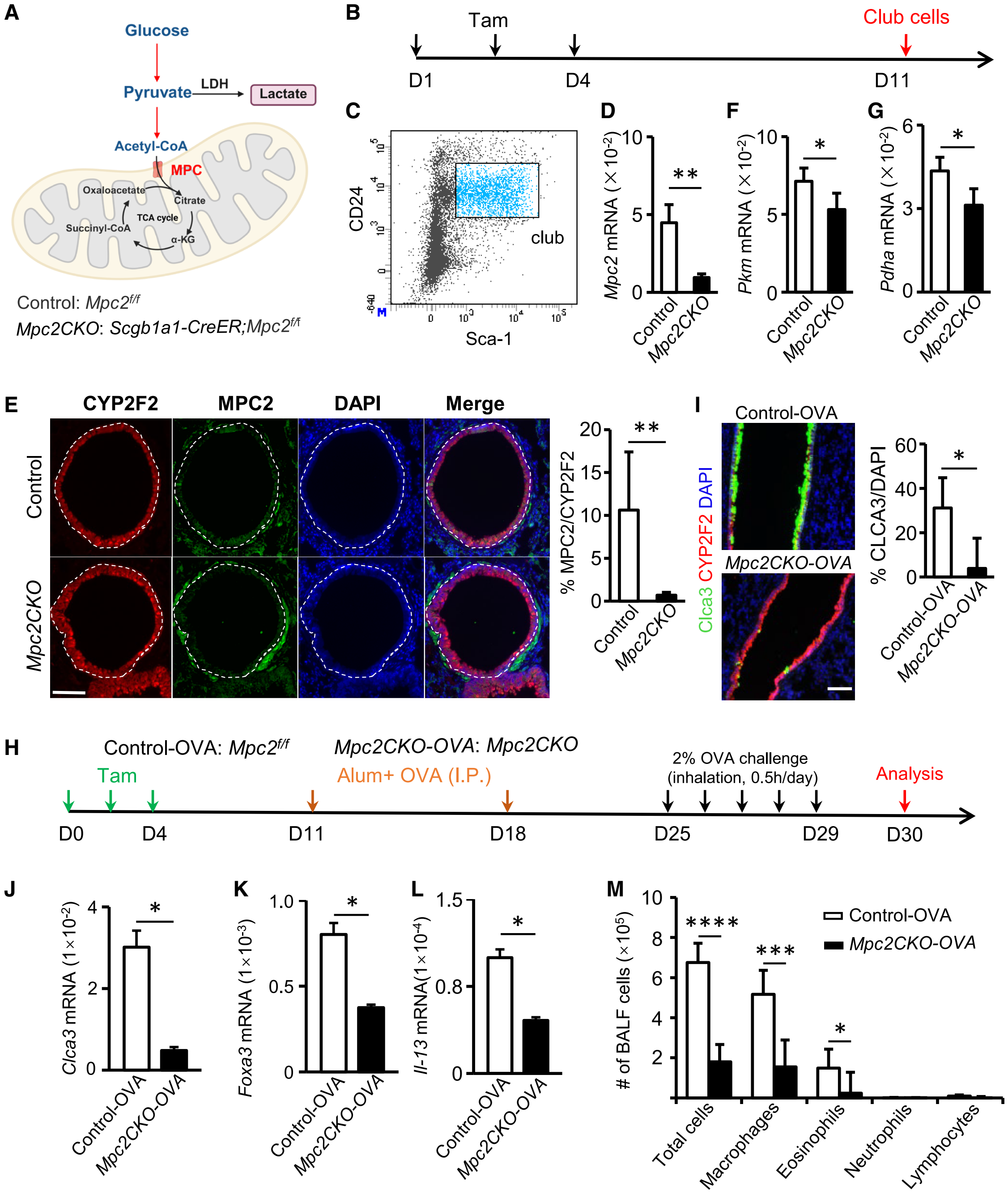

Figure 2. Mitochondrial pyruvate metabolism promotes the differentiation of club cells into goblet cells

(A) Schematic illustration showing the role of the mitochondrial pyruvate carrier 1/2 (MPC1/2) in transporting pyruvate into mitochondria for oxidative phosphorylation. Conditional knockout mice with targeted deletion of *Mpc2* in club cells (*Scgb1a1-CreER;Mpc2*$^{f/f}$, referred to as *Mpc2CKO*) were generated by crossing *Scgb1a1-CreER* mice with *Mpc2*$^{f/f}$ mice. The littermate *Mpc2*$^{f/f}$ mice served as the control group (control). The schematic was generated in BioRender.

(B) To induce specific deletion of *Mpc2* in club cells, control and *Mpc2CKO* mice were intraperitoneally injected with Tam on days 1 and 4. Lung tissues were harvested on day 11 for downstream analyses.

(C) Club cells from lung tissue were isolated using FACS. After excluding debris based on forward and side scatter (FSC/SSC), viable cells were selected by DAPI-negative staining. Epithelial cells were identified as EpCAM-positive and negative for CD31, CD34, and CD45. Club cells were further gated based on dual positivity for CD24 and Sca-1.

(legend continued on next page)

control and dimensionality reduction of scRNA-seq data, cell populations were grouped into four major categories: epithelial, stromal, endothelial, and immune cells (Figures 4B and S3). Differential gene expression analysis using the FindMarkers function revealed 16 upregulated and 1 downregulated gene in club cells from *Mpc2CKO-OVA* mice compared to control-OVA. To validate these findings, we also performed bulk RNA-seq on whole lung tissue and intersected the differentially expressed genes (DEGs) with those identified in scRNA-seq. This yielded four overlapping genes (Figure 4C), among which *Cxcl17* and *Wfdc2* were preferentially expressed in epithelial cells (Figure 4D). Further analysis showed that *Cxcl17* was upregulated in both club and goblet cells, while *Wfdc2* was elevated primarily in club cells (Figure 4E). Similar results were confirmed in the bulk RNA-seq dataset (Figure 4F). To determine whether *Cxcl17* upregulation was dependent on OVA-induced inflammation or reflected a baseline effect of MPC2 deletion, we examined lungs from *Mpc2CKO* mice in the absence of OVA challenge. qPCR analysis of sorted club cells revealed that *Cxcl17* expression was already significantly higher in *Mpc2CKO* mice compared with control (Figure 4G). These data indicate that loss of *Mpc2* intrinsically enhances *Cxcl17* expression in club cells even under baseline conditions. Importantly, re-analysis of human scRNA-seq datasets demonstrated that *CXCL17* expression was also significantly higher in club cells from asthmatic patients compared with non-asthmatic control (Figure 4H). These results suggest that *Cxcl17* expression is more sensitive to alterations in the pyruvate oxidative phosphorylation pathway, and its upregulation may be a key downstream effect of *Mpc2* deletion in secretory epithelial cells.

Mpc2 knockdown in club cells enhances interaction with alveolar macrophages via the *Cxcl17-Cxcr4* signaling axis

To further investigate how *Mpc2* deficiency in club cells leads to reduced airway inflammation, we conducted cell-cell communication analysis using CellChat based on the scRNA-seq data. We observed an increased number of ligand-receptor interactions between club cells and macrophages in the *Mpc2CKO-OVA* group (Figure 5A). Notably, the interaction between *Cxcl17* (ligand) and *Cxcr4* (receptor) was significantly enhanced, suggesting that club cells may regulate macrophage function via the *Cxcl17-Cxcr4* axis (Figure 5B). We then performed deeper sub-clustering of macrophages, identifying seven distinct subtypes, including alveolar macrophages (AMΦ), precursors (Pro_AMΦ and Pro_IMΦ), monocyte-derived macrophages (MoMΦ), and three transitional or regulatory subtypes (Folr2$^+$ IMΦ, Folr2$^-$Spp1$^-$ IMΦ, and Spp1$^+$ IMΦ) (Figures 5C and S4). DEG analysis revealed that alveolar macrophages exhibited the largest number of transcriptional changes between control-OVA and *Mpc2CKO-OVA* groups (Figure 5D), suggesting that they are the primary target of club-cell-derived signals. Specifically, *Ccl17*, associated with type 2 immune responses, was significantly downregulated in alveolar macrophages from the *Mpc2CKO-OVA* group (Figure 5E). *Ccl17* was minimally expressed in other macrophage subtypes (Figure 5F).

To validate this, we performed an intervention experiment using a CCL17-neutralizing antibody in the OVA-induced asthma model (Figure 6A). Compared with the OVA and isotype control groups, mice treated with the anti-CCL17 antibody showed significantly reduced total BALF cells, particularly eosinophils (Figure 6B). Lung tissue expression of *Il-13* and *Foxa3* was also significantly decreased (Figures 6C and 6D). These results provide causal evidence that CCL17 is a downstream effector of the *Cxcl17-Cxcr4* signaling axis and that its neutralization effectively mitigates airway inflammation, consistent with the phenotype observed in *Mpc2CKO* mice. Taken together, these findings support a model in which disruption of pyruvate oxidative phosphorylation in club cells leads to the upregulation of *Cxcl17*, which subsequently signals through the CXCR4 receptor on alveolar macrophages. This interaction suppresses the expression of

(D) qPCR analysis of *Mpc2* mRNA expression in sorted club cells. Data are presented as mean ± SD, with $n = 6$ per group. Statistical significance was determined by two-tailed unpaired Student's t test; $^{**}p < 0.01$.

(E) Representative immunofluorescence staining of lung sections showing MPC2 (green) and CYP2F2 (red) in control and *Mpc2CKO* mouse airways. The white dashed circles delineate the regions of CYP2F2+ club cells. Quantification of MPC2/CYP2F2 cells is shown on the right. Data are presented as mean ± SD, with $n = 6$ per group. Scale bars, 50 μm, $^{**}p < 0.01$.

(F and G) qPCR analysis of *Pkm* and *Pdha* expression in sorted club cells. Data are presented as mean ± SD, with $n = 6$ per group. Statistical significance was determined by two-tailed unpaired Student's t test; $^{*}p < 0.05$.

(H) Experimental timeline for the OVA-induced allergic airway inflammation model. *Mpc2*$^{f/f}$ (control-OVA, $n = 6$) and *Scgb1a1-CreER; Mpc2*$^{f/f}$ (*Mpc2CKO-OVA*, $n = 6$) mice were treated with Tam and OVA aerosol before tissue and BALF collection.

(I) Effects of *Mpc2* deletion on goblet cell differentiation. Immunofluorescence of lung sections showing CLCA3$^+$ goblet cells (scale bars, 50 μm).

(J–L) qPCR analysis of *Clca3* (J), *Foxa3* (K), and *Il-13* (L) mRNA levels in lung tissue. qPCR analysis of expression in lung tissues. Data are shown as mean ± SD. $^{*}p < 0.05$.

(M) Hema 3 staining of BALF cells showing eosinophils, neutrophils, and macrophages. Data are shown as mean ± SD. $^{*}p < 0.05$, $^{***}p < 0.001$, $^{****}p < 0.0001$.

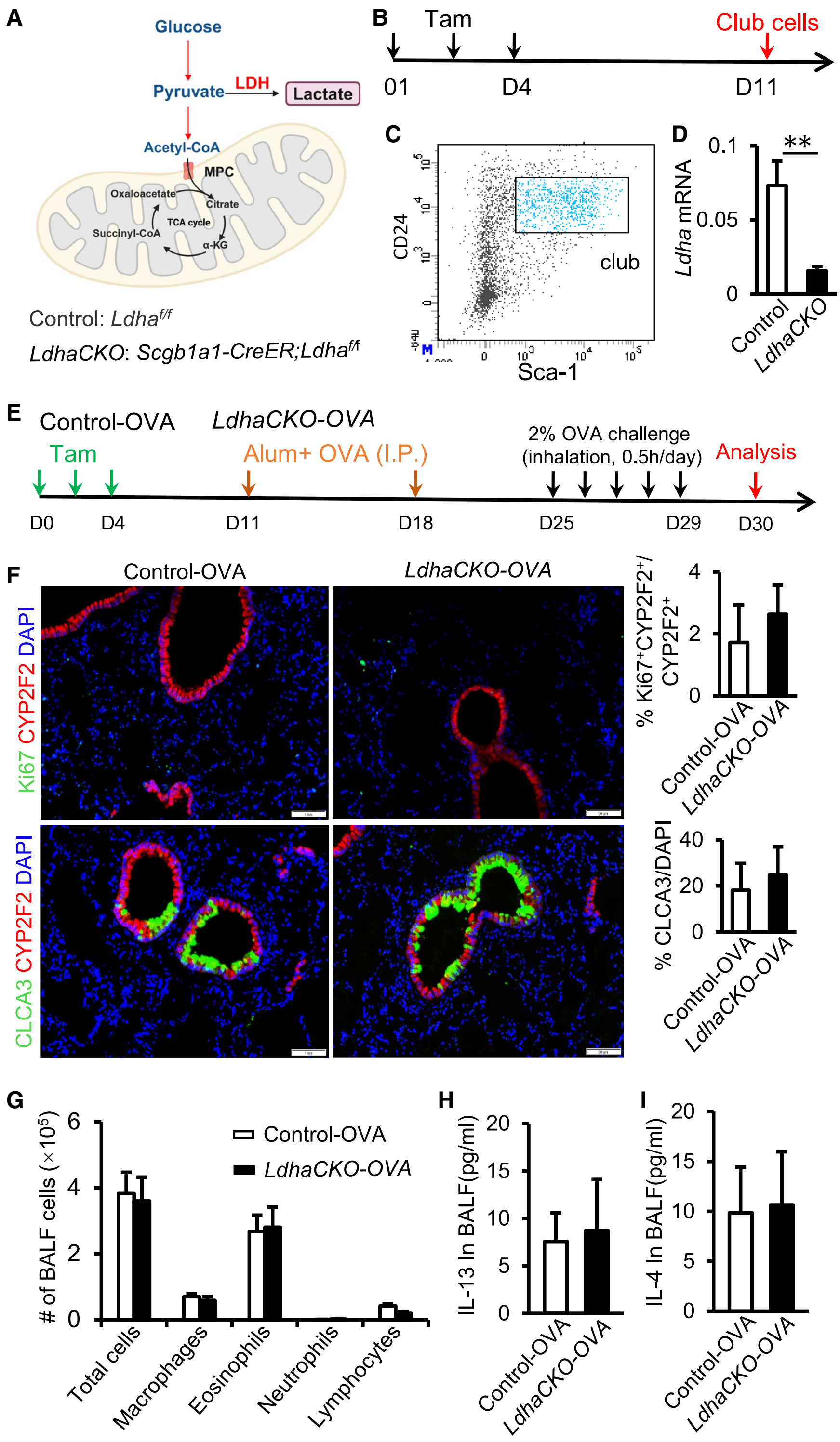

Figure 3. Club-to-goblet cell differentiation is independent of the pyruvate-lactate pathway

(A) Pyruvate is converted into lactate in the cytoplasm by LDH. The schematic was generated in BioRender.

(B) Experimental design schematic. *Ldha*$^{f/f}$ mice (control) and *Scgb1a1-CreER; Ldha*$^{f/f}$ mice (*LdhaCKO*) were intraperitoneally injected with Tam to induce conditional knockout of the *Ldha* gene specifically in club cells. Tam was administered on day 1 and day 4, and lung tissues were collected on day 11 for subsequent analyses.

(C) Club cells were isolated from lung single-cell suspensions using FACS. After removing debris based on FSC/SSC and selecting live cells (DAPI-negative), epithelial cells were identified by EpCAM positivity and negative expression of CD31, CD34, and CD45. Club cells were finally sorted based on double positivity for Sca-1 and CD24.

(D) qPCR was performed on sorted club cells to assess *Ldha* mRNA expression levels. Data are presented as mean ± SD, with $n = 6$ per group.

(E) Experimental design for OVA-induced allergic airway inflammation model using control (control-OVA, $n = 8$) and *LdhaCKO* mice (*LdhaCKO-OVA*, $n = 8$), with Tam and OVA treatments.

(F) Immunofluorescence analysis shows no significant difference in club cell proliferation or goblet cell (CLCA3+) differentiation between control-OVA and *LdhaCKO-OVA* groups (scale bars 50 μm).

(G) Hema 3 staining and differential cell counting of BALF were performed to characterize immune cell subtypes.

(H and I) IL-4 (H) and IL-13 (I) concentrations in BALF were quantified using ELISA. Data are presented as mean ± SD. Statistical significance was assessed by unpaired two-tailed Student's *t* test (**$p < 0.01$).

pro-inflammatory chemokines, particularly CCL17, thereby reducing Th2 cytokine production, limiting eosinophil recruitment, and ultimately attenuating allergic airway inflammation (Figure 6E).

DISCUSSION

This study reveals the critical role of pyruvate metabolic reprogramming in airway club cells in the context of

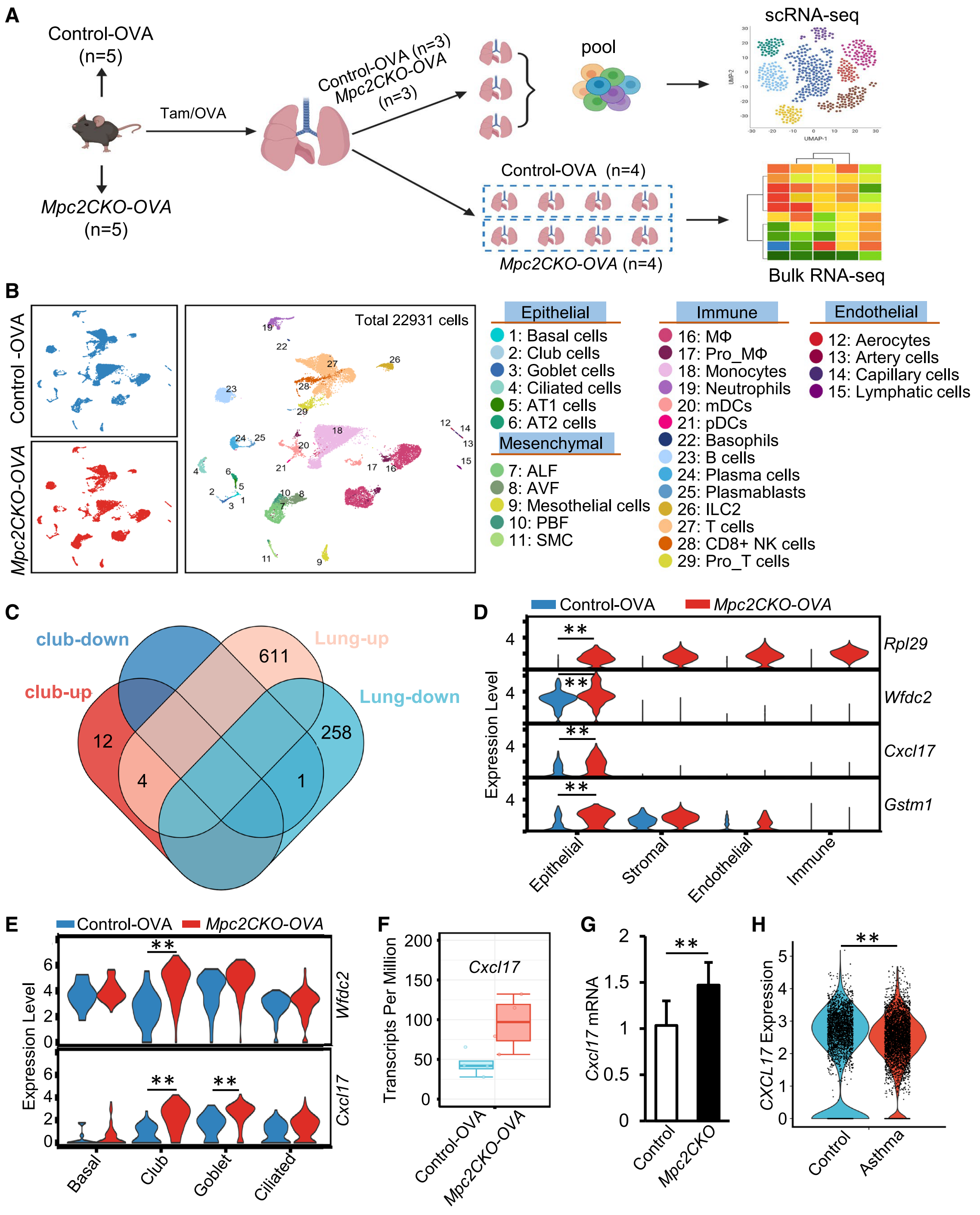

Figure 4. MPC2 knockout remodels the transcriptional landscape of club cells

(A) The schematic illustrates the experimental workflow for OVA-induced allergic airway inflammation model in *Mpc2* conditional knockout mice. Mice were divided into two groups: control-OVA ($n = 5$) and *Mpc2CKO-OVA* ($n = 5$). All mice received Tam and OVA treatment to induce airway inflammation. Lung tissues were collected to prepare single-cell suspensions for both scRNA-seq and whole-lung bulk RNA-seq analyses. For scRNA-seq, three mice from each group were randomly selected, and 20,000 viable cells (viability >90%) were extracted from each mouse. Equal volumes of cell suspensions from mice in the same group were pooled to generate two independent samples for high-throughput sequencing. For bulk RNA-seq, individual libraries were constructed and sequenced for all mice. The control-OVA and *Mpc2CKO-OVA* groups each yielded four qualified samples ($n = 4$). The schematic was generated in BioRender.

(legend continued on next page)

asthma-associated airway inflammation and remodeling. Specifically, we suggest that the oxidative phosphorylation pathway of pyruvate is essential for the differentiation and function of club cells. Our results show, for the first time, that glycolytic activity in airway club cells is significantly increased in asthma patients, suggesting that abnormal pyruvate metabolism may be a key pathological mechanism in asthma progression. Using a conditional knockout mouse model with club-cell-specific deletion of the *Mpc2* gene, we found that the loss of MPC2 significantly suppressed the differentiation of club cells into goblet cells and alleviated OVA-induced airway inflammation. Moreover, *Mpc2* deletion led to elevated expression of the chemokine *Cxcl17* in club cells, which enhanced the interaction between club cells and alveolar macrophages. These findings indicate that metabolic pathways not only supply cellular energy but also play essential roles in immune signaling.

Our observation that glycolysis is elevated in club and goblet cells in asthma is consistent with previous studies. Prior research has shown that airway epithelial cells in asthma exhibit enhanced glycolysis (van de Wetering et al., 2021) and that inhibiting glycolysis in OVA-induced mouse model significantly reduces airway inflammation and hyperresponsiveness (Zhao et al., 2017). Pyruvate, a product of glycolysis, can either enter mitochondria via the MPC complex for oxidative phosphorylation or be converted into lactate via LDH. We showed that *Mpc2* deletion markedly inhibited club cell differentiation into goblet cells, aligning with earlier findings where blocking oxidative phosphorylation in intestinal crypt epithelial cells reduced goblet cell differentiation (Sunderhauf et al., 2021). Additionally, we found that *Mpc2*-deficient club cells led to reduced OVA-induced airway inflammation and an increase in ligand-receptor interactions between club cells and macrophages—most notably, the *Cxcl17-Cxcr4* axis, suggesting that *Mpc2* deletion may modulate macrophage function via this pathway and thereby attenuate inflammation.

Cxcl17 is a member of the CXCL chemokine family (Pisabarro et al., 2006; Weinstein et al., 2006) and has been reported to suppress inflammatory injury by recruiting myeloid-derived suppressor cells (Oka et al., 2017; Zhang et al., 2025). It can bind CXCR4 in a dose-dependent manner, competitively inhibiting other pro-inflammatory chemokines (Giblin et al., 2023; White et al., 2024). Our study suggests that *Cxcl17* is upregulated in *Mpc2*-deficient club cells and acts on macrophages via the *Cxcl17-Cxcr4* axis, leading to macrophage suppression and reduced release of *Ccl17*. Previous studies have shown that CXCL17 suppresses *CCL24* via the p38 mitogen-activated protein kinase pathway to prevent eosinophilic airway inflammation (Zhang et al., 2018), while CCL17 can promote Th2 cytokine (IL-13) expression through a positive feedback loop (Barnes, 2008; Nicolas et al., 2024). These reports are consistent with our findings that *Ccl17* production in alveolar macrophages and *Il-13* production in Th2 cells were both reduced in *Mpc2*-deficient mice. Consistent with this model, CCL17 neutralization during OVA challenge further attenuated airway inflammation, as shown by reduced total cell counts and eosinophils in BALF and by decreased lung *Il-13* and *Foxa3* expression. These results provide functional support for the *Cxcl17-Cxcr4-Ccl17* signaling axis (Figures 6B–6D).

This study is the first systematic demonstration of the dual role of oxidative phosphorylation in club cells in influencing differentiation programs and modulating the immune microenvironment. Previous research on airway progenitor cells has mainly focused on signaling pathways, with little attention to metabolic regulation. Our study proposes a novel model in which metabolic-immune signaling regulates airway inflammation. Additionally, we reveal a previously underappreciated immunomodulatory role of CXCL17 under conditions of metabolic imbalance, broadening our understanding of this chemokine in asthma pathogenesis and offering a theoretical foundation for the development of metabolism-targeted therapies.

However, several limitations should be acknowledged. First, due to the limited number of club cells in the murine airway, we were unable to directly measure oxidative

(B) Single-cell transcriptomic analysis of mouse lung tissue. UMAP plots illustrate the distribution of various cell subtypes in lung samples from control-OVA and *Mpc2CKO-OVA* mice.

(C) Venn diagram of DEGs in club cells and whole lung tissue. “club-down” and “club-up” represent downregulated and upregulated genes, respectively, in club cells from scRNA-seq data (control-OVA vs. *Mpc2CKO-OVA*), while “Lung-down” and “Lung-up” refer to genes downregulated and upregulated in bulk RNA-seq analysis of whole lung tissue (control-OVA vs. *Mpc2CKO-OVA*).

(D) Expression patterns of four representative upregulated genes identified in (C) within the club cell cluster, indicating their marked activation following *Mpc2* deletion.

(E) Expression of two characteristic epithelial-related genes across different lung cell populations, further illustrating the impact of gene deletion on cell fate determination.

(F) Expression changes of *Cxcl17* in lung tissue based on bulk RNA-seq data.

(G) qPCR analysis of *Cxcl17* expression in sorted club cells isolated from lungs of *Mpc2CKO* and control mice without OVA challenge. Data are presented as mean ± SD; $n = 6$ per group; $^{**}p < 0.01$.

(H) Violin plots showing *CXCL17* expression in human club cells from publicly available scRNA-seq datasets. $^{**}p < 0.01$.

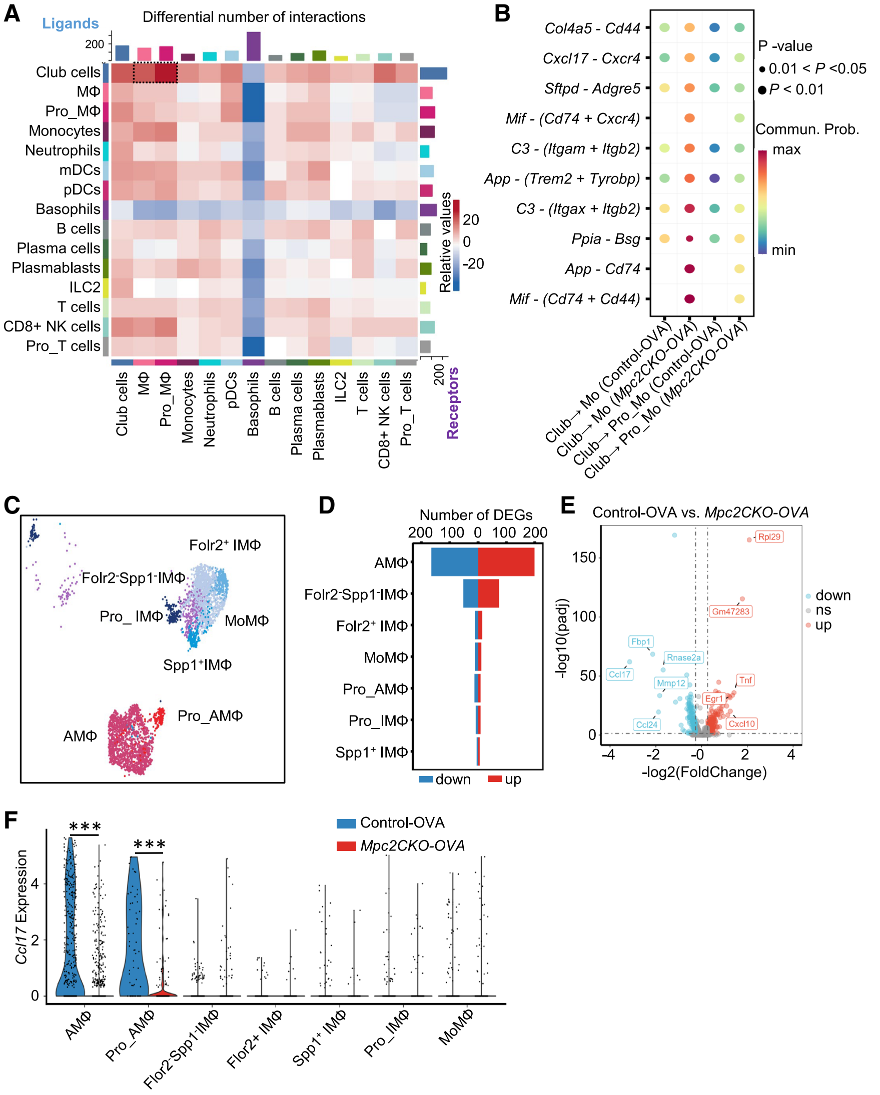

Figure 5. Club-cell-derived *Cxcl17* suppresses *Ccl17* expression in alveolar macrophages

(A) Using the CellChat tool to analyze integrated single-cell transcriptomic data, we reconstructed the intercellular signaling network within the lung and generated a heatmap to illustrate differences in the number of ligand-receptor interactions between club cells and immune cells.

(B) Ligand-receptor pairs between club cells and macrophages, including proliferating macrophages.

(C) Clustering analysis of lung macrophage populations identified seven distinct subclusters, including alveolar macrophages (AMΦ), precursor subsets (Pro_AMΦ and Pro_IMΦ), monocyte-derived macrophages (MoMΦ), and several intermediate immunoregulatory macrophage states (Folr2^{+} IMΦ, Folr2^{-}Spp1^{-} IMΦ, and Spp1^{+} IMΦ). Their distribution was visualized using an UMAP plot.

(D) The number of DEGs in each macrophage subpopulation was quantified. Compared to the control-OVA group, downregulated genes in the *Mpc2CKO-OVA* group are shown in blue, and upregulated genes are shown in red. A two-group comparison (control-OVA vs. *Mpc2CKO-OVA*) was performed, and DEGs were identified based on the criteria of $\log_2$(fold change) > 1.5 and adjusted $p < 0.05$ (Benjamini-Hochberg correction).

(E) The volcano plot displays differentially expressed genes in the AMΦ (alveolar macrophage) population of *Mpc2CKO-OVA* mice compared to the control-OVA group. Downregulated genes in the *Mpc2CKO-OVA* group are shown in blue, while upregulated genes are shown in red.

(F) The violin plot shows the expression levels of *Ccl17* across different macrophage subpopulations.

phosphorylation activity, which weakens the direct mechanistic evidence. Future studies could overcome this limitation by using airway organoid cultures or human induced pluripotent stem cell-derived models. Second, the role of the *Cxcl17-Cxcr4* axis was mainly inferred through transcriptomic and ligand-receptor analyses and lacks direct experimental validation, such as co-culture or neutralizing antibody assays. Future work should strengthen mechanistic validation. Third, this study used the OVA sensitization/challenge model, which—while reproducible for type 2 airway inflammation—does not capture the full complexity of natural aeroallergens (e.g., house dust mite [HDM]). We therefore interpret our findings alongside convergent evidence from human scRNA-seq and *in vivo* CCL17 neutralization. Future work will employ HDM to test whether the MPC2-dependent *Cxcl17-Ccl17* pathway also operates under more physiological allergen exposure. Finally, this study was limited to mouse models; thus, the clinical relevance of our findings remains to be confirmed. Further studies using human lung tissues or clinical cohorts are warranted to enhance translational significance.

METHODS

Mice

All animal procedures were reviewed and approved by the Institutional Animal Care and Use Committee of Haihe

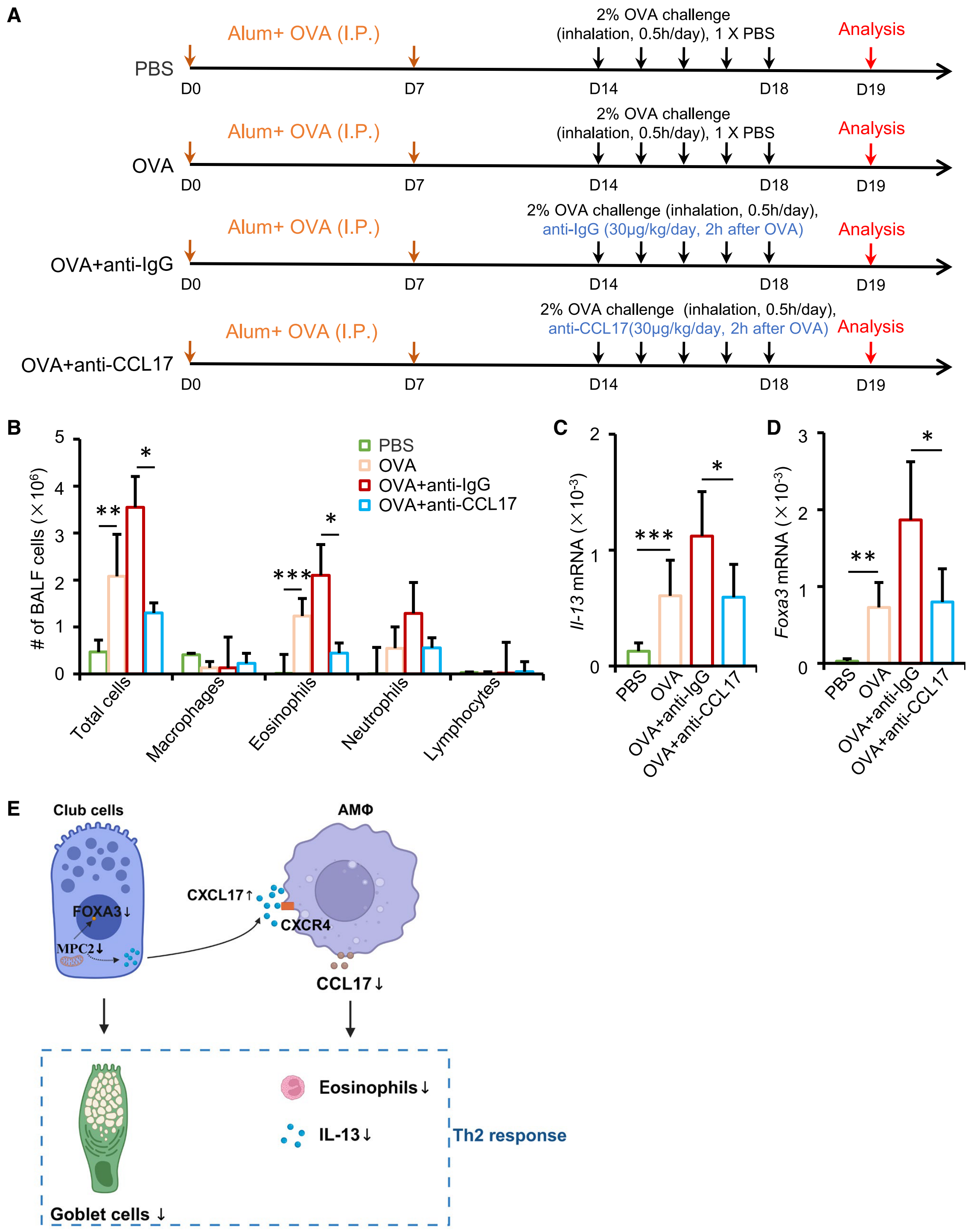

Figure 6. CCL17 neutralization attenuates OVA-induced mouse airway inflammation in mice

(A) Experimental design of the OVA-induced allergic airway inflammation model and anti-CCL17 neutralization treatment. Mice were sensitized with Alum + OVA by intraperitoneal injection on days 0 and 7, followed by 2% OVA aerosol challenges for 30 min daily from days 14 to 18. Anti-CCL17 antibody (30 μg/kg/day) or control IgG was administered via transoral instillation 2 h after each OVA challenge. Samples were collected 24 h after the final challenge.

(B) Hema 3 staining and differential cell counting of BALF were performed to characterize immune cell subtypes (n = 6 per group; mean ± SD; *$p < 0.05$, **$p < 0.01$, ***$p < 0.001$).

(legend continued on next page)

Hospital, Tianjin University. All mice were housed under specific pathogen-free conditions in the animal facility of Haihe Hospital (License no.: SYXK [Jin] 2021-0002). *Ldha*$^{f/f}$, *Mpc2*$^{f/f}$, and *Scgb1a1-CreER* mice were obtained from Jackson Laboratory. *Scgb1a1-CreER; Ldha*$^{f/f}$ and *Scgb1a1-CreER; Mpc2*$^{f/f}$ mice were generated by crossing *Ldha*$^{f/f}$ or *Mpc2*$^{f/f}$ mice with *Scgb1a1-CreER* mice. To induce gene deletion in club cells, 6- to 8-week-old mice were intraperitoneally injected with Tam at 200 mg/kg every other day for a total of three injections. Mice were maintained for 1 week after the final injection before subsequent experiments. All mice used in this study were on a C57BL/6 background. Mice were housed under a 12 h light/12 h dark cycle at a room temperature of 19°C–25°C, following the guidelines of the Guide for the Care and Use of Laboratory Animals.

OVA-induced allergic airway inflammation model in transgenic mice

The model was established according to previously published protocols (Li et al., 2019, 2022). Female mice (6–8 weeks old) were sensitized by intraperitoneal injection of 0.1 mL of 10 mg/mL OVA (Sigma-Aldrich) emulsified in 44% $Al(OH)_3$ (Thermo Fisher Scientific) on days 11 and 18 after the final Tam injection. From days 25 to 29, mice were challenged by ultrasonic nebulization of 2% OVA solution (w/v) for 30 min daily. On day 30, mice were euthanized, and lung tissues and BALF were collected for analysis.

Preparation of single-cell suspensions

The protocol for preparing single-cell suspensions was adapted from our previous publications (Cheng et al., 2024; Wang et al., 2020b). Mice were anesthetized with 1% pentobarbital sodium, and lungs were perfused with 1× PBS to collect BALF. Cardiac perfusion with 1× PBS was performed, and the lungs were excised and injected five times with elastase solution (Worthington Biochemical Corporation) (first injection: 1 mL, subsequent injections: 0.5 mL each at 5-min intervals). Lungs were minced and digested with DNase I (Sigma-Aldrich) at 37°C for 15 min. The cell suspension was filtered through a 70 μm cell strainer, centrifuged at 700*g* for 8 min, and treated with 1 mL red blood cell lysis buffer for 90 s. The resulting single-cell suspension was used for scRNA-seq and bulk transcriptome sequencing (bulk RNA-seq).

BALF from OVA-challenged mice

BALF was collected from OVA-challenged mice. Samples were centrifuged at 300 × *g* for 10 min at 4°C to separate the supernatant and cells. The supernatant was used for ELISA assays, while the cell pellet was prepared into cytospin slides. The slides were stained using Hema 3 staining (Fisherbrand) to analyze infiltrating leukocytes, including eosinophils, neutrophils, and macrophages.

ELISA

Levels of IL-4 and IL-13 in BALF supernatants were measured using ELISA kits (Abcam) according to the manufacturer's instructions.

FACS of club cells

FACS of club cells was performed as described previously (Wang et al., 2020b; Zhao et al., 2022). Single-cell suspensions were incubated with primary antibodies: anti-mouse CD24, Ly-6A/E (Sca1), PE/Cyanine-labeled EpCAM, CD31 (PECAM-1), CD34, CD45, and 7-AAD viability dye at 4°C for 1 h. After washing, secondary antibody (streptavidin APC-eFluor 780) was added and incubated at 4°C for another hour. Cells were sorted using an FACS Aria III cell sorter (BD Biosciences). Club cells were identified as $CD31^-CD34^-CD45^-EpCAM^+Sca1^+CD24^+$ (Yue et al., 2023).

scRNA-seq library construction and sequencing

Single-cell libraries were prepared using the SeekOne Digital Droplet 3′ Single-Cell Library Preparation Kit (SeekGene) and the SeekOne Digital Droplet System. This system enabled the entire workflow from single-cell RNA capture and reverse transcription to library construction. First, cell viability and concentration of lung single-cell suspensions were assessed using a Countstar cell counter, ensuring cell viability exceeded 90%. Then, 10,000 cells were mixed with reverse transcription reagents and loaded into the sample well of the SeekOne chip. Barcoded hydrogel beads (BHBs) and oil phase were subsequently added to

(C and D) qPCR analysis of *Il-13* and *Foxa3* mRNA levels in lung tissue (n = 6 per group). Data are presented as mean ± SD. Statistical significance was determined using one-way ANOVA followed by Tukey's multiple-comparison test (*$p < 0.05$, **$p < 0.01$, ***$p < 0.001$).
(E) This diagram summarizes the proposed mechanism by which Mpc2 regulates airway inflammation. Under asthmatic conditions, glycolytic activity in airway club cells is enhanced, promoting their differentiation into goblet cells and leading to increased goblet cell metaplasia. Deletion of *Mpc2* in club cells impairs the mitochondrial oxidative metabolism of pyruvate, resulting in decreased expression of *Foxa3* and subsequent inhibition of goblet cell generation. Meanwhile, MPC2 deficiency enhances the secretion of the chemokine *Cxcl17* by club cells, which binds to its receptor *Cxcr4* on macrophages, strengthening the interaction between the two cell types. This interaction suppresses the pro-inflammatory functions of macrophages and reduces the secretion of *Ccl17*. Collectively, these changes lead to decreased recruitment of type 2 inflammatory cells and a marked alleviation of allergic airway inflammation. The schematic was generated in BioRender.

designated wells to generate water-in-oil droplets. Within each droplet, single cells interacted with BHBs to capture RNA.

Reverse transcription was performed at 42°C for 90 min, followed by enzyme inactivation at 80°C for 15 min. After the reaction, emulsions were broken to extract and purify cDNA, which was then amplified by PCR. The amplified cDNA underwent fragmentation, end repair, and adapter ligation, followed by purification using DNA magnetic beads (Vazyme). Library quality was assessed using Qubit (Thermo Fisher Scientific) and BioFragment Analyzer (Bioptic Qsep400). Libraries were required to meet quality criteria: concentration ≥1 ng/μL and fragment size between 350 and 750 bp; libraries containing short fragments were re-purified. Qualified libraries were sequenced on the Illumina NovaSeq 6000 platform using PE150 mode. The target sequencing depth was more than 50,000 reads per cell to ensure high-quality single-cell transcriptome data.

scRNA-seq data analysis

Quality control of raw FASTQ files generated by the Illumina NovaSeq 6000 platform was performed using FastQC to assess read quality, GC content, and adapter contamination. Low-quality reads and adapter sequences were removed using Trim Galore to ensure high-quality data for downstream analysis. Transcript alignment and gene expression quantification were performed using CellRanger software. Cells with fewer than 200 detected genes or with mitochondrial gene content exceeding 20% were excluded. Cells with transcript counts between 500 and 5,000 genes were retained. The gene expression matrix was normalized using the NormalizeData function in the Seurat R package. The "LogNormalize" method was applied to log-transform expression values and scale them based on total expression. Highly variable genes were identified using the FindVariableFeatures function in Seurat for downstream dimensionality reduction and clustering. Principal-component analysis was performed to extract major feature vectors. Uniform manifold approximation and projection (UMAP) was then applied for nonlinear dimensionality reduction and visualization of cell clusters in low-dimensional space. Clustering analysis was performed using the FindClusters function, and cell types were annotated based on known marker gene expression.

Bulk RNA-seq

Cell viability of the single-cell suspensions was assessed by trypan blue exclusion, ensuring a viability rate above 90%. Total RNA was extracted using TRIzol reagent (Invitrogen), and RNA concentration was measured with a NanoDrop 2000 spectrophotometer (Thermo Fisher Scientific). RNA quality was evaluated using an Agilent 2100 Bioanalyzer, with samples requiring an RNA integrity number > 7.0 for downstream sequencing. High-quality RNA was reverse transcribed into cDNA using the SMART-Seq v.4 Ultra Low Input RNA Kit. Between 1 and 10 ng of total RNA was used for first-strand synthesis, followed by PCR amplification to obtain sufficient cDNA. Library construction was performed using the Illumina TruSeq Stranded mRNA Library Prep Kit, including mRNA enrichment, cDNA end repair, adapter ligation, and PCR amplification. Library quality was assessed by qPCR to ensure suitability for sequencing. Qualified libraries were sequenced using the Illumina NovaSeq 6000 platform with 150 bp paired-end reads. Raw data were quality checked with FastQC, and low-quality sequences and adapters were removed. Reads were aligned to the mouse reference genome using STAR (v.2.7.9a) or CellRanger (v.6.0.0). Expression matrices were generated and analyzed using Seurat (v.4.0) or Scanpy (v.1.9), and differential gene expression was identified. Enrichment analyses were conducted using Gene Ontology (GO) and Kyoto Encyclopedia of Genes and Genomes (KEGG) to explore the biological functions of DEGs.

Gene Expression Omnibus data analysis

Public scRNA-seq datasets were retrieved from the Gene Expression Omnibus (GEO). Based on these searches, three datasets were selected: GSE164015, GSE193816, and GSE143868. The first two datasets included samples from asthma patients, while GSE143868 included healthy controls. To ensure consistent alignment, all datasets used the GRCh38 human genome reference. Data from GSE143868 were extracted from an integrated human lung single-cell atlas. Subsequent analysis was performed in R (v.4.2.1) using the Seurat package. Quality control included retaining cells with ≥200 expressed genes and filtering genes expressed in ≥3 cells. Mitochondrial gene percentage thresholds were determined dynamically using violin plots. Doublets were removed using the DoubletFinder package. After merging samples, data were normalized, and highly variable genes were selected. Technical noise, including mitochondrial gene expression, was regressed out via linear regression. Dimensionality reduction was performed using PCA, and batch effects were corrected using the Harmony package. The number of principal components was determined using elbow plots. UMAP was applied to visualize clusters, and clustering was done with the Louvain algorithm (resolution = 2). Cell type annotation was based on canonical marker genes, verified independently by two researchers, with classification of both major populations and subtypes.

Immunostaining

Mouse lung tissues were fixed in 4% paraformaldehyde for 2 h, followed by dehydration in graded ethanol and paraffin embedding. Tissue sections (4 μm thick) were prepared for staining. Antigen retrieval was performed in

10 mM buffer for 20 min. Sections were blocked with 5% bovine serum albumin in PBS and incubated with primary antibodies overnight at 4°C. After washing, sections were incubated with secondary antibodies at 37°C for 2 h. Nuclei were counterstained with DAPI in PBS for 5 min, and slides were sealed with mounting medium. Fluorescence images were captured using a fluorescence microscope with consistent exposure and filter settings, and signal intensity was quantified accordingly.

RNA isolation and gene expression analysis

Total RNA was extracted from sorted airway epithelial cells, lung tissues, or organoids using TRIzol reagent (Invitrogen). One microgram of RNA was reverse transcribed into cDNA using the HiScript III RT SuperMix Kit (Vazyme). qPCR was performed on a Roche LightCycler 96 system using 100 ng cDNA and ChamQ Universal SYBR qPCR Master Mix (Vazyme). The qPCR protocol included initial denaturation at 95°C for 2 min, followed by 35 cycles of 95°C for 10 s, 60°C for 20 s, and 72°C for 15 s. Gene expression levels were calculated using the $2^{-\Delta\Delta Ct}$ method, normalized to GAPDH as the internal control.

CCL17 antibody instillation

Female C57BL/6 mice (6–8 weeks old) were divided into four groups (PBS, OVA, OVA + anti-IgG, and OVA + anti-CCL17; n = 6 per group). Mice were intraperitoneally sensitized with alum-precipitated OVA (20 μg OVA + 2 mg alum in 200 μL PBS) on days 0 and 7 and challenged with 2% OVA aerosol (0.5 h per day) on days 14–18 using a nebulizer. Control animals received PBS instead of OVA during the challenges. For antibody intervention, a neutralizing antibody against CCL17 (30 μg/kg/day; R&D Systems) or isotype IgG control was administered via transoral instillation (2 h after each OVA aerosol challenge). Mice were sacrificed 24 h after the final OVA challenge (day 19). Lung tissues and BALF were collected for analysis.

Statistical analysis

All experiments were independently repeated three times, and data are presented as mean ± standard deviation (SD). Comparisons between groups were performed using two-tailed unpaired Student's t tests. For comparisons involving three or more groups, one-way ANOVA followed by Tukey's multiple-comparison test was used. For single-cell transcriptomic data, differential gene expression analysis between cell clusters was performed using the Wilcoxon rank-sum test. DEGs were identified based on the criteria of $\log_2$(fold change) > 1.5 and an adjusted $p < 0.05$, corrected using the Benjamini-Hochberg method. Pathway enrichment analysis of DEGs was conducted using the clusterProfiler package to perform GO and KEGG analyses, with a significance threshold of false discovery rate < 0.05. In addition, cell-cell communication analysis was carried out using the CellChat package by computing interaction strengths of ligand-receptor pairs and assessing statistical significance of differences using permutation tests. Statistical significance was defined as $*p < 0.05$, $**p < 0.01$, and $***p < 0.001$, $****p < 0.0001$.

RESOURCE AVAILABILITY

Lead contact

Further information and requests for resources, reagents, and materials should be directed to and will be fulfilled by the lead contact, Huaiyong Chen (huaiyong.chen@foxmail.com).

Materials availability

This study did not generate new unique reagents. All mouse strains used are available upon reasonable request from the corresponding author.

Data and code availability

All newly generated sequencing data in this study will be deposited in the China National Gene Bank Sequence Archive (CNSA: CNP0008222; https://db.cngb.org). Publicly available single-cell RNA sequencing datasets used for validation were retrieved from GEO: GSE164015, GSE193816, and GSE143868. No new custom code was generated in this study; however, the scripts used for data analysis are available from the corresponding author upon reasonable request.

ACKNOWLEDGMENTS

This work was supported by the National Natural Science Foundation of China (no. 82570001 and 82070001), Ministry of Science and Technology of the People's Republic of China (no. 2024YFA1108906 and 2023YFC3502605), the Tianjin Key Medical Discipline (Specialty) Construction Project (no. TJYXZDXK-063B), the Key Laboratory of Medical Rescue Key Technology and Equipment, Ministry of Emergency Management (Open Fund Project no. YJBKFKT202410), the Tianjin Key Medical Discipline Construction Project (no. TJYXZDXK-3-018B), the Second Batch of High-Level Talent Training Program for the Health Industry in Tianjin (no. TJSQNYXXR-D2-070), and the Natural Science Foundation of Tianjin (no. 25JCZDJC01260).

AUTHOR CONTRIBUTIONS

Conceptualization, J.W.; methodology, J.W. and C.D.; investigation, J.W. and C.D.; formal analysis, D.H.; data curation, D.H. and Q.W.; resources, Y.L., K.L., X.L., and Q.Z.; visualization, B.G. and L.L.; validation, C.D.; statistical analysis, B.G.; data interpretation, Y.L., K.L., X.L., and Q.Z.; writing – original draft, J.W.; writing – review and editing, D.H., L.L., and H.C.; supervision, H.C.; project administration, H.C.; funding acquisition, H.C., J.W., and L.L.

DECLARATION OF INTERESTS

The authors declare no competing interests.

SUPPLEMENTAL INFORMATION

Supplemental information can be found online at https://doi.org/10.1016/j.stemcr.2025.102742.

Received: April 27, 2025
Revised: November 14, 2025
Accepted: November 14, 2025
Published: December 18, 2025

REFERENCES

Barcik, W., Boutin, R.C.T., Sokolowska, M., and Finlay, B.B. (2020). The Role of Lung and Gut Microbiota in the Pathology of Asthma. Immunity *52*, 241–255. https://doi.org/10.1016/j.immuni.2020.01.007.

Barnes, P.J. (2008). The cytokine network in asthma and chronic obstructive pulmonary disease. J. Clin. Investig. *118*, 3546–3556. https://doi.org/10.1172/JCI36130.

Bondeelle, L., Clement, S., Bergeron, A., and Tapparel, C. (2025). Lung stem cells and respiratory epithelial chimerism in transplantation. Eur. Respir. Rev. *34*, 240146. https://doi.org/10.1183/16000617.0146-2024.

Bradding, P., Porsbjerg, C., Côté, A., Dahlén, S.E., Hallstrand, T.S., and Brightling, C.E. (2024). Airway hyperresponsiveness in asthma: The role of the epithelium. J. Allergy Clin. Immunol. *153*, 1181–1193. https://doi.org/10.1016/j.jaci.2024.02.011.

Cheng, P., Chen, Y., Wang, J., Han, Z., Hao, D., Li, Y., Feng, F., Duan, X., and Chen, H. (2024). PM(2.5) induces a senescent state in mouse AT2 cells. Environ. Pollut. *347*, 123686. https://doi.org/10.1016/j.envpol.2024.123686.

Flores, A., Schell, J., Krall, A.S., Jelinek, D., Miranda, M., Grigorian, M., Braas, D., White, A.C., Zhou, J.L., Graham, N.A., et al. (2017). Lactate dehydrogenase activity drives hair follicle stem cell activation. Nat. Cell Biol. *19*, 1017–1026. https://doi.org/10.1038/ncb3575.

Giblin, S.P., Ranawana, S., Hassibi, S., Birchenough, H.L., Mincham, K.T., Snelgrove, R.J., Tsuchiya, T., Kanegasaki, S., Dyer, D., and Pease, J.E. (2023). CXCL17 binds efficaciously to glycosaminoglycans with the potential to modulate chemokine signaling. Front. Immunol. *14*, 1254697. https://doi.org/10.3389/fimmu.2023.1254697.

Hamanaka, R.B., Glasauer, A., Hoover, P., Yang, S., Blatt, H., Mullen, A.R., Getsios, S., Gottardi, C.J., DeBerardinis, R.J., Lavker, R.M., and Chandel, N.S. (2013). Mitochondrial reactive oxygen species promote epidermal differentiation and hair follicle development. Sci. Signal. *6*, ra8. https://doi.org/10.1126/scisignal.2003638.

Heijink, I.H., Kuchibhotla, V.N.S., Roffel, M.P., Maes, T., Knight, D.A., Sayers, I., and Nawijn, M.C. (2020). Epithelial cell dysfunction, a major driver of asthma development. Allergy *75*, 1902–1917. https://doi.org/10.1111/all.14421.

Li, K., Li, M., Li, W., Yu, H., Sun, X., Zhang, Q., Li, Y., Li, X., Li, Y., Abel, E.D., et al. (2019). Airway epithelial regeneration requires autophagy and glucose metabolism. Cell Death Dis. *10*, 875. https://doi.org/10.1038/s41419-019-2111-2.

Li, K., Zhang, Q., Li, L., Li, S., Yue, Q., Li, X., Li, Y., Wang, J., Wang, S., Yuan, Z., and Chen, H. (2022). DJ-1 governs airway progenitor cell/eosinophil interactions to promote allergic inflammation. J. Allergy Clin. Immunol. *150*, 1178–1193.e13. https://doi.org/10.1016/j.jaci.2022.03.036.

Li, Y., He, Y., Zheng, Q., Zhang, J., Pan, X., Zhang, X., Yuan, H., Wang, G., Liu, X., Zhou, X., et al. (2025). Mitochondrial pyruvate carriers control airway basal progenitor cell function through glycolytic-epigenetic reprogramming. Cell Stem Cell *32*, 105–120.e6. https://doi.org/10.1016/j.stem.2024.09.015.

Ly, C.H., Lynch, G.S., and Ryall, J.G. (2020). A Metabolic Roadmap for Somatic Stem Cell Fate. Cell Metab. *31*, 1052–1067. https://doi.org/10.1016/j.cmet.2020.04.022.

Nicolas, L.S.S., Czarnowicki, T., Akdis, M., Pujol, R.M., Lozano-Ojalvo, D., Leung, D.Y.M., Guttman-Yassky, E., and Santamaria-Babi, L.F. (2024). CLA+ memory T cells in atopic dermatitis. Allergy *79*, 15–25. https://doi.org/10.1111/all.15816.

Oka, T., Sugaya, M., Takahashi, N., Takahashi, T., Shibata, S., Miyagaki, T., Asano, Y., and Sato, S. (2017). CXCL17 Attenuates Imiquimod-Induced Psoriasis-like Skin Inflammation by Recruiting Myeloid-Derived Suppressor Cells and Regulatory T Cells. J. Immunol. *198*, 3897–3908. https://doi.org/10.4049/jimmunol.1601607.

Pisabarro, M.T., Leung, B., Kwong, M., Corpuz, R., Frantz, G.D., Chiang, N., Vandlen, R., Diehl, L.J., Skelton, N., Kim, H.S., et al. (2006). Cutting edge: novel human dendritic cell- and monocyte-attracting chemokine-like protein identified by fold recognition methods. J. Immunol. *176*, 2069–2073. https://doi.org/10.4049/jimmunol.176.4.2069.

Shao, S., Zhang, N., Specht, G.P., You, S., Song, L., Fu, Q., Huang, D., You, H., Shu, J., Domissy, A., et al. (2024). Pharmacological expansion of type 2 alveolar epithelial cells promotes regenerative lower airway repair. Proc. Natl. Acad. Sci. USA *121*, e2400077121. https://doi.org/10.1073/pnas.2400077121.

Sunderhauf, A., Hicken, M., Schlichting, H., Skibbe, K., Ragab, M., Raschdorf, A., Hirose, M., Schaffler, H., Bokemeyer, A., Bettenworth, D., et al. (2021). Loss of Mucosal p32/gC1qR/HABP1 Triggers Energy Deficiency and Impairs Goblet Cell Differentiation in Ulcerative Colitis. Cell. Mol. Gastroenterol. Hepatol. *12*, 229–250. https://doi.org/10.1016/j.jcmgh.2021.01.017.

van de Wetering, C., Manuel, A.M., Sharafi, M., Aboushousha, R., Qian, X., Erickson, C., MacPherson, M., Chan, G., Adcock, I.M., ZounematKermani, N., et al. (2021). Glutathione-S-transferase P promotes glycolysis in asthma in association with oxidation of pyruvate kinase M2. Redox Biol. *47*, 102160. https://doi.org/10.1016/j.redox.2021.102160.

Varricchi, G., Brightling, C.E., Grainge, C., Lambrecht, B.N., and Chanez, P. (2024). Airway remodelling in asthma and the epithelium: on the edge of a new era. Eur. Respir. J. *63*, 2301619. https://doi.org/10.1183/13993003.01619-2023.

Wang, J., Li, K., Hao, D., Li, X., Zhu, Y., Yu, H., and Chen, H. (2024). Pulmonary fibrosis: pathogenesis and therapeutic strategies. MedComm *5*, e744. https://doi.org/10.1002/mco2.744.

Wang, J., Li, X., and Chen, H. (2020a). Organoid models in lung regeneration and cancer. Cancer Lett. *475*, 129–135. https://doi.org/10.1016/j.canlet.2020.01.030.

Wang, J., Li, X., Wang, A., Zhao, F., Wu, Q., Li, L., Yu, H., Wu, J., and Chen, H. (2020b). Organoid technology demonstrates effects of potential drugs for COVID-19 on the lung regeneration. Cell Prolif. *53*, e12928. https://doi.org/10.1111/cpr.12928.

Wang, X., Su, S., Zhu, Y., Cheng, X., Cheng, C., Chen, L., Lei, A., Zhang, L., Xu, Y., Ye, D., et al. (2023). Metabolic Reprogramming via ACOD1 depletion enhances function of human induced pluripotent stem cell-derived CAR-macrophages in solid tumors. Nat. Commun. *14*, 5778. https://doi.org/10.1038/s41467-023-41470-9.

Weinstein, E.J., Head, R., Griggs, D.W., Sun, D., Evans, R.J., Swearingen, M.L., Westlin, M.M., and Mazzarella, R. (2006). VCC-1, a novel chemokine, promotes tumor growth. Biochem. Biophys. Res. Commun. *350*, 74–81. https://doi.org/10.1016/j.bbrc.2006.08.194.

White, C.W., Platt, S., Kilpatrick, L.E., Dale, N., Abhayawardana, R.S., Dekkers, S., Kindon, N.D., Kellam, B., Stocks, M.J., Pfleger, K.D.G., and Hill, S.J. (2024). CXCL17 is an allosteric inhibitor of CXCR4 through a mechanism of action involving glycosaminoglycans. Sci. Signal. *17*, eabl3758. https://doi.org/10.1126/scisignal.abl3758.

Yue, Q., Li, K., Song, Z., Wang, Q., Wang, J., Li, X., Li, Y., Zhang, Q., Zhu, Y., and Chen, H. (2023). Nitric oxide hinders club cell proliferation through Gdpd2 during allergic airway inflammation. FEBS Open Bio. *13*, 1041–1055. https://doi.org/10.1002/2211-5463.13617.

Zhang, K., Liang, Y., Feng, Y., Wu, W., Zhang, H., He, J., Hu, Q., Zhao, J., Xu, Y., Liu, Z., and Zhen, G. (2018). Decreased epithelial and sputum miR-221-3p associates with airway eosinophilic inflammation and CXCL17 expression in asthma. Am. J. Physiol. Lung Cell. Mol. Physiol. *315*, L253–L264. https://doi.org/10.1152/ajplung.00567.2017.

Zhang, S., Sun, Z., Chen, Z., Bi, Y., Wei, S., Mao, Z., Jin, J., Ding, Y., and Wang, W. (2025). Endothelial YAP/TEAD1-CXCL17 signaling recruits myeloid-derived suppressor cells against liver ischemia-reperfusion injury. Hepatology *81*, 888–902. https://doi.org/10.1097/HEP.0000000000000773.

Zhao, B., Shi, C., Wang, X., Sun, Z., Ruan, Y., Wang, X., Zhang, Z., Xie, T., Shan, J., Wang, J., and Qian, G. (2025). Kechuan Decoction mitigates apoptosis of airway epithelial cells by improving lipid metabolism disorders and mitochondria dysfunction in HDM-induced asthma. Phytomedicine *136*, 156299. https://doi.org/10.1016/j.phymed.2024.156299.

Zhao, F., Wang, J., Wang, Q., Hou, Z., Zhang, Y., Li, X., Wu, Q., and Chen, H. (2022). Organoid technology and lung injury mouse models evaluating effects of hydroxychloroquine on lung epithelial regeneration. Exp. Anim. *71*, 316–328. https://doi.org/10.1538/expanim.21-0168.

Zhao, Q., Chu, Z., Zhu, L., Yang, T., Wang, P., Liu, F., Huang, Y., Zhang, F., Zhang, X., Ding, W., and Zhao, Y. (2017). 2-Deoxy-d-Glucose Treatment Decreases Anti-inflammatory M2 Macrophage Polarization in Mice with Tumor and Allergic Airway Inflammation. Front. Immunol. *8*, 637. https://doi.org/10.3389/fimmu.2017.00637.

Zhu, L., An, L., Ran, D., Lizarraga, R., Bondy, C., Zhou, X., Harper, R.W., Liao, S.Y., and Chen, Y. (2019). The Club Cell Marker SCGB1A1 Downstream of FOXA2 is Reduced in Asthma. Am. J. Respir. Cell Mol. Biol. *60*, 695–704. https://doi.org/10.1165/rcmb.2018-0199OC.

Stem Cell Reports, Volume *21*

Supplemental Information

Mitochondrial pyruvate metabolism in club cells drives airway inflammation

Jianhai Wang, Chunnan Du, De Hao, Qian Wu, Biyu Gui, Yu Li, Kuan Li, Xue Li, Qiuyang Zhang, Li Li, and Huaiyong Chen

Supplemental Information

Mitochondrial pyruvate metabolism in club cells drives airway inflammation

Jianhai Wang, Chunnan Du, De Hao, Qian Wu, Biyu Gui, Yu Li, Kuan Li, Xue Li, Qiuyang Zhang, Li Li, Huaiyong Chen

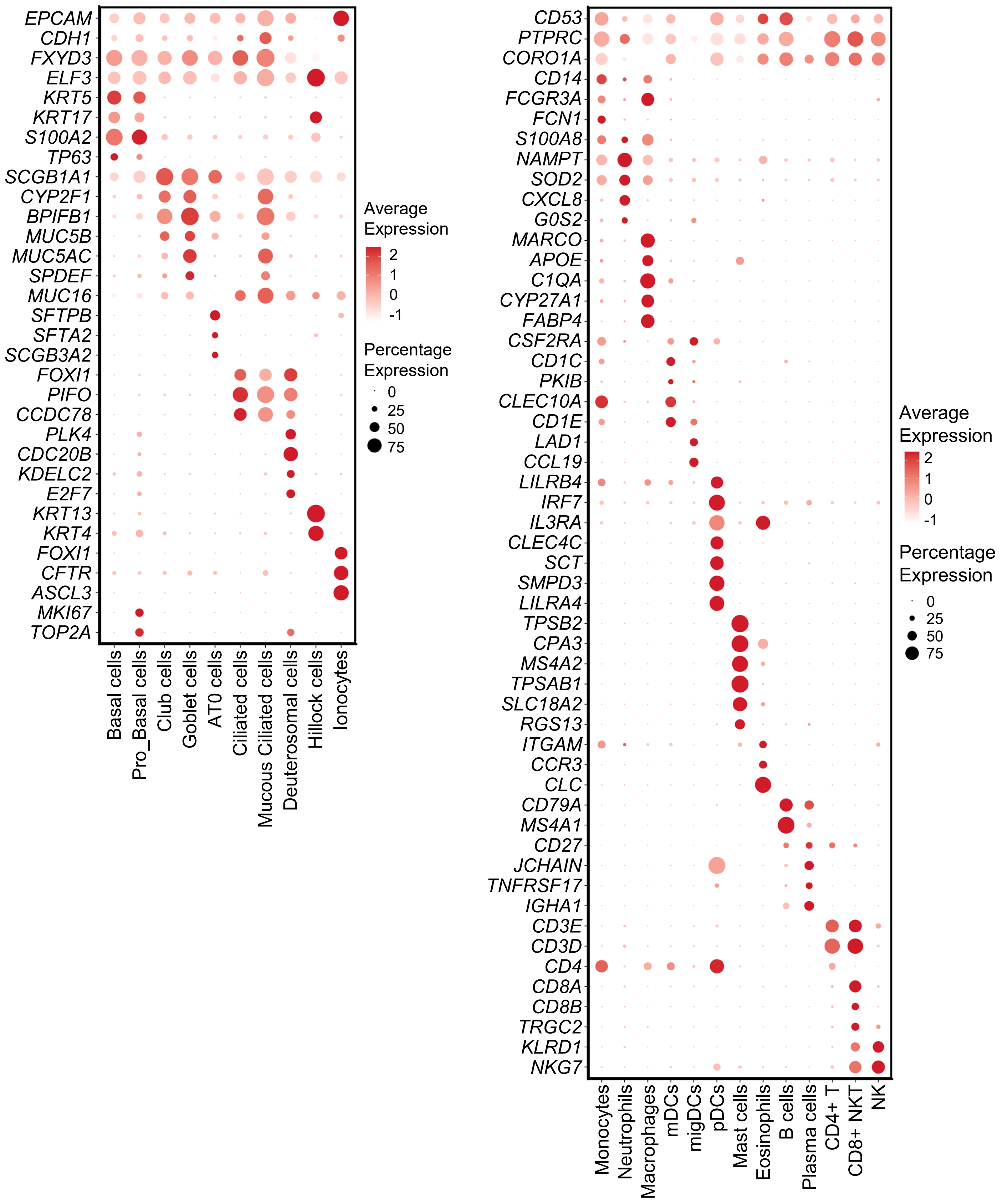

Figure S1. Marker gene expression patterns across distinct cell types

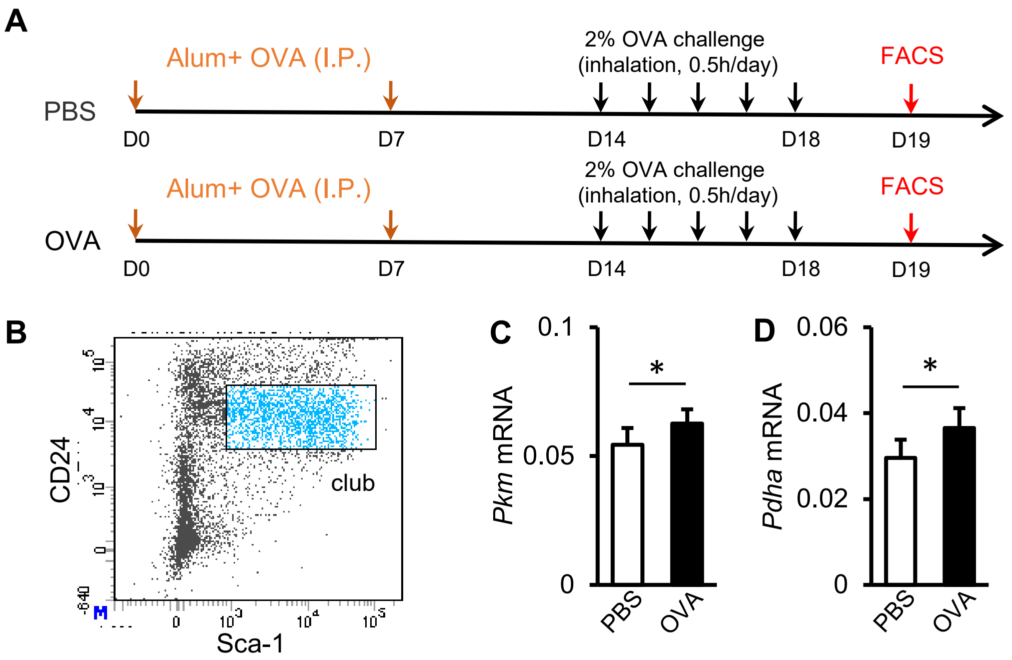

Figure S2. OVA-induced inflammation activates glycolysis and OXPHOS in club cells.

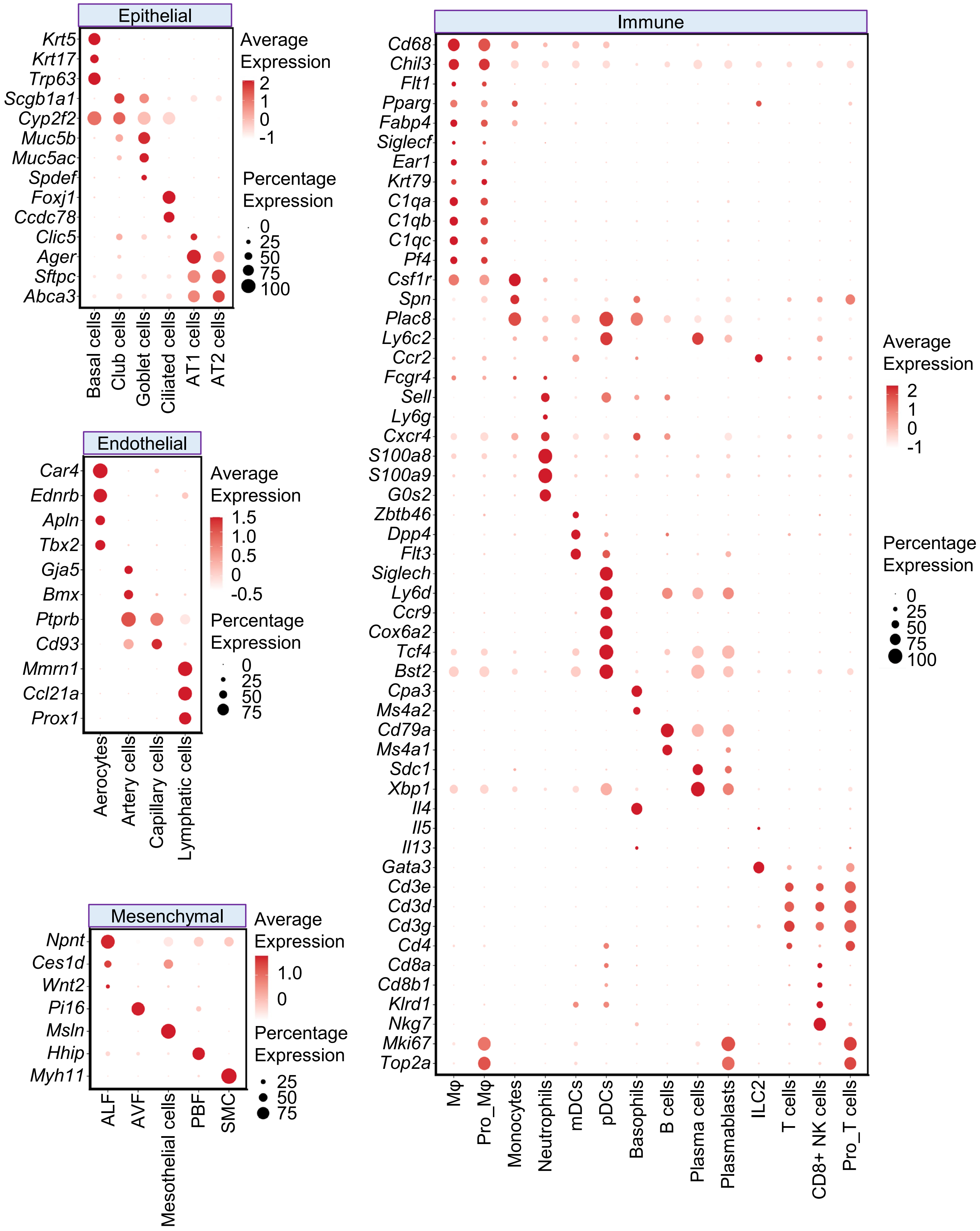

Figure S3. Marker gene expression patterns across distinct cell types.

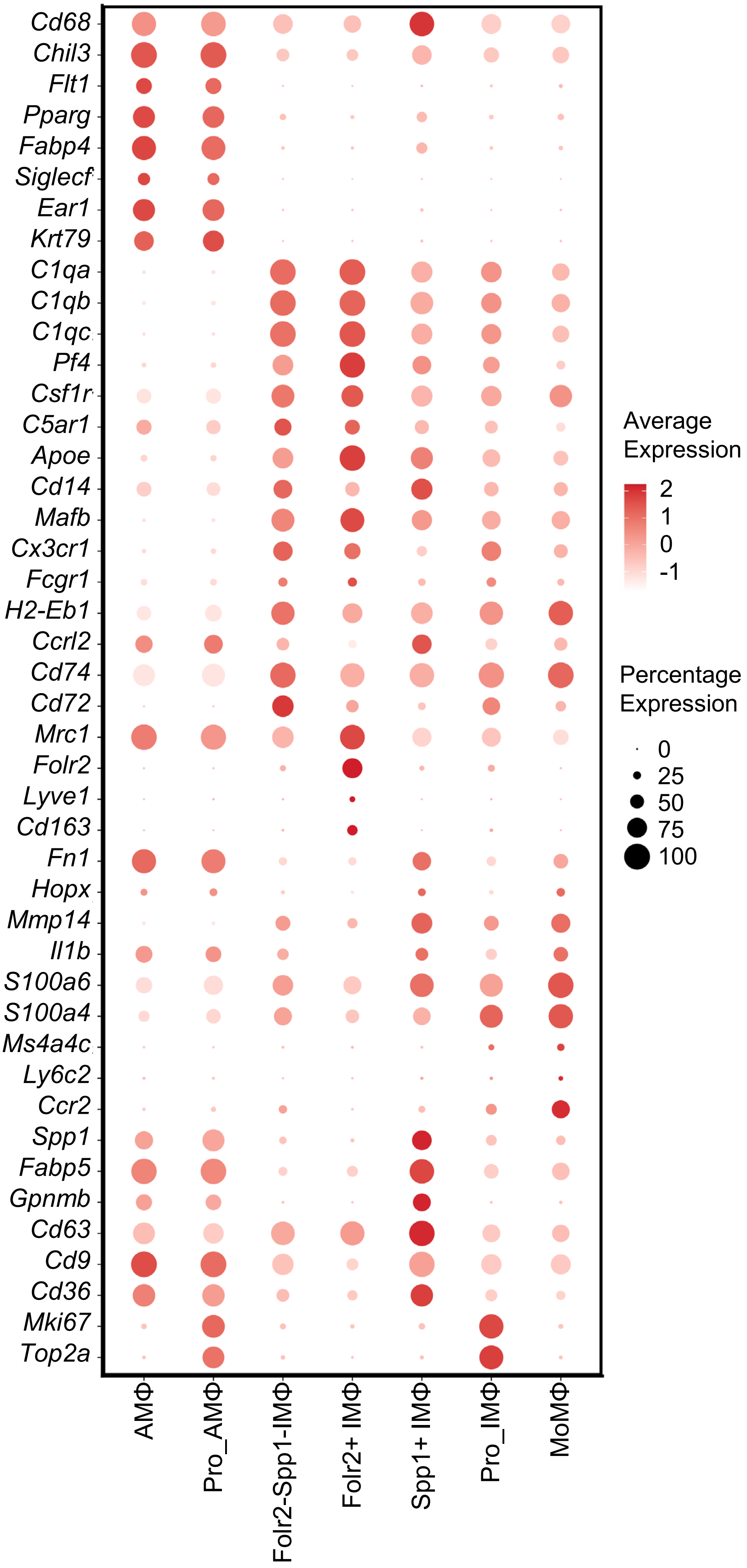

Figure S4. Marker gene expression patterns of macrophages.

Figure S1 Marker gene expression patterns across distinct cell types

The dot plot illustrates the average expression levels (color intensity) and the proportion of positively expressing cells (dot size) for representative marker genes across 23 cell types. The left panel displays marker expression in epithelial-derived cells (e.g., EPCAM, KRT5, SCGB1A1, FOXJ1, PIFO), while the right panel shows marker expression in immune cell populations (e.g., CD14, FCGR3A, MARCO, IL3RA, CD3D, CD79A, NKG7). Color indicates the average expression value.

Figure S2 OVA-induced inflammation activates glycolysis and OXPHOS in club cells

(A) Schematic illustration of the OVA-induced asthma model. Mice were sensitized by intraperitoneal injection of alum/OVA on days 0 and 7, followed by exposure to 2% OVA aerosol for 30 minutes per day from days 14 to 18. Lung tissues were harvested 24 hours after the final challenge (day 19).

(B) Representative flow cytometry plot showing the gating strategy for isolating $CD24^{+}Sca\text{-}1^{+}$ club cells from lung tissue.

(C - D) qPCR was performed on sorted club cells from OVA-treated mice compared with PBS controls ($P < 0.05$, n = 5 per group) to assess *Pkm* mRNA (C) and *Pdha* mRNA expression (D) levels. Data are presented as mean ± SD; statistical significance was determined using an unpaired two-tailed Student's t-test.

Figure S3 Marker gene expression patterns across distinct cell types

Dot plot showing expression of canonical marker genes in epithelial, endothelial, mesenchymal, and immune cell types. Dot color reflects average expression level, while size indicates the percentage of cells expressing each gene. Marker genes distinguish key subtypes such as club, goblet, and AT2 cells; endothelial and fibroblast subpopulations; and diverse immune cell types including macrophages, neutrophils, dendritic cells, and T/B cells.

Figure S4 Marker gene expression patterns of macrophages

A dot plot of marker genes across macrophage subpopulations reveals distinct transcriptional signatures. The color gradient represents the average expression level (Average Expression), with lighter to darker shades indicating increasing expression.

The size of each dot indicates the percentage of cells within the cluster that positively express the corresponding gene.

Supplemental experimental methods

The OVA-induced model was conducted according to the protocol described in the main text, with identical dose, timeline, and treatments. Cell sorting and molecular assays were performed as described in the “Flow cytometry” and “qPCR” sections of the main text. The qPCR target genes were *Pkm* and *Pdha*. Relative expression was calculated using the $2^{-\Delta\Delta Ct}$ method with Gapdh as the reference gene. Data are presented as mean ± SD. Group comparisons were performed using unpaired two-tailed Student’s t-tests with a significance threshold of $P < 0.05$. Biological replicates were n = 5 per group.
